# Magnification-independent breast cancer diagnosis using a GWO-enhanced vision transformer with multi-stage stain normalization

**DOI:** 10.1038/s41598-026-42490-3

**Published:** 2026-03-04

**Authors:** Taiyaba Fatma, Prabhat Kumar Sahu, Sasanka Choudhury, Aneesh Wunnava

**Affiliations:** 1 Department of Computer Science and Engineering, Siksha ‘O’ Anusandhan (Deemed to be University), Bhubaneswar, Odisha India; 2 Department of Computer Science and Information Technology, Siksha ‘O’ Anusandhan (Deemed to be University), Bhubaneswar, Odisha India; 3 Department of Mechanical Engineering, Siksha ‘O’ Anusandhan (Deemed to be University), Bhubaneswar, Odisha India; 4 Department of Electronics and Communication Engineering, Siksha ‘O’ Anusandhan (Deemed to be University), Bhubaneswar, Odisha India

**Keywords:** BreakHis dataset, Vision transformer (ViT), Grey wolf optimizer (GWO), Stain normalization, Data augmentation, Magnification-invariant modeling, Deep learning, Cancer, Computational biology and bioinformatics, Engineering, Mathematics and computing

## Abstract

Breast cancer diagnosis from histopathological images remains a critical yet challenging task due to staining variability, magnification differences, and complex tissue morphology. This study presents a comprehensive preprocessing, balancing, and classification framework for the BreakHis dataset, integrating advanced stain normalization, magnification-wise augmentation, and an optimized Vision Transformer architecture. A four-stage normalization pipeline comprising CLAHE, histogram matching, Shades-of-Gray correction, and Macenko stain normalization was developed to standardize color distribution and enhance structural clarity across 7909 images. To address severe class imbalance, a targeted magnification-specific augmentation strategy expanded the dataset to 11,848 images with equal benign and malignant representation. A Vision Transformer (ViT) model was designed for each of the four magnifications (40X, 100X, 200X, 400X), and further optimized using the Grey Wolf Optimizer (GWO) to automatically tune hyperparameters such as transformer depth, attention heads, embedding dimensions, dropout, and learning rate. Experimental results demonstrate high and consistent performance across magnifications, with accuracies of 92.1%, 92.9%, 93.5%, and 94.0%, respectively. Cross-validation reveals minimal variance, confirming model robustness and generalization. The proposed GWO-ViT framework establishes a reliable, magnification-invariant, and computationally efficient solution for automated breast cancer histopathology classification, offering strong potential for clinical integration.

## Introduction

Breast cancer is still a widespread and dangerous illness globally, remaining a significant health issue because many people get it, it has different forms, and deaths from it are increasing. According to recent worldwide cancer reports, breast cancer now has more new cases each year than any other cancer, with millions diagnosed and a large number of women dying from it. Early detection is critical because survival rates are significantly higher when the disease is identified in its initial stages, allowing clinicians to provide personalized and effective treatment strategies. Histopathological examination of biopsy tissue is the clinical gold standard for diagnosing breast cancer, as it allows detailed evaluation of cellular morphology, structural abnormalities, and tumor invasiveness. Nevertheless, making diagnoses by hand using microscopes demands a lot of effort, takes a significant amount of time, and can differ based on who is looking at the samples, especially when slight variations in tissue need to be spotted using different levels of magnification.

Artificial intelligence has significantly improved the automation of medical imaging processes, especially for tasks involving image classification, segmentation, and object detection. Within AI, deep learning has become a dominant approach for developing modern computer-aided diagnosis (CAD) systems^1,2^.In the past, the majority of computer-aided detection (CAD) systems designed to identify breast cancer have used convolutional neural networks (CNNs) as the primary structural component. Rapid advancement of deep neural networks that use Transformer architecture, first widely adopted in the field of natural language processing, has led to adjustments for use in computer vision, which has resulted in the development of Vision Transformers (ViTs). Designed specifically for visual understanding, ViTs effectively handle tasks including classification, segmentation, and object detection. They have demonstrated strong performance across various benchmark datasets and often outperform CNN-based models. A major strength of Vision Transformers is their minimal dependence on built-in inductive biases; unlike CNNs, they do not rely on predefined assumptions about local spatial patterns or translation consistency^3,4^. Approaches grounded in transfer learning remain highly prevalent for scrutinizing breast cancer imagery, with considerable research underscoring that refining pre-existing models holds the capacity to substantially boost efficacy, notably in situations where data accessibility is restricted. Assessments contrasting diverse structural designs have brought to light that the extent of network layering and the methods employed in preprocessing exert a considerable impact on the precision of categorization, with models like Exception and DarkNet frequently exhibiting satisfactory performance even when augmentation strategies are kept to a bare minimum. Hybrid and ensemble approaches have further enhanced diagnostic reliability by combining features at multiple scales or aggregating predictions from complementary models^5^. Beyond end-to-end deep learning, several works have integrated handcrafted descriptors—such as texture, shape, and color features—with deep feature extractors to improve multi-class classification across magnification levels. Using techniques to pull out significant attributes and subsequently implement standard categorization tools - for example, random forests, closest-neighbor systems, or gradient enhancing models has led to remarkable results, thus highlighting the sustained importance of combined strategies in producing precise and dependable breast cancer identification^6,7^.

Beyond breast cancer histopathology, deep learning–based diagnostic frameworks have been successfully applied to a wide range of oncological imaging modalities. Ho et al.^8^ demonstrated the feasibility of deep learning–driven cancer detection using sub-micron full-field optical coherence tomography (FF-OCT) images for squamous cell carcinoma, highlighting the potential of noninvasive, high-resolution imaging combined with automated analysis. Similarly, Sun et al.^9^ proposed an attention-based convolutional neural network (HIENet) for endometrial cancer diagnosis from histopathological images, achieving strong classification performance and improved interpretability through attention mechanisms. In the context of breast cancer, Karthiga and Narashimhan^10^ explored deep convolutional neural networks and transfer learning strategies for histopathology image classification, reporting competitive accuracy for both binary and multi-class settings. These studies collectively underscore the growing importance of deep learning techniques in histopathological cancer diagnosis while also revealing persistent challenges related to stain variability, magnification dependence, and robust feature generalization—challenges that motivate the development of the proposed GWO-enhanced Vision Transformer framework. Collectively, these investigations highlight noteworthy patterns in the analysis of histopathological images: a prevalent dependence on transfer learning methodologies, an amplified integration of both hybrid and ensemble approaches, a burgeoning focus on classification methods unaffected by magnification levels, and the sustained significance of manually designed features. However, obstacles persist in relation to the expense of computation, the comprehensibility of models, inconsistencies in magnification, and the capacity to generalize across different histopathology facilities, thereby encouraging the creation of more sophisticated hybrid frameworks.

### Research objectives

The primary objectives of this research are:

- To create a cutting-edge deep learning system designed for classifying breast cancer histopathology images, capable of combining both detailed, localized features and broad, comprehensive characteristics.
- To maintain consistent effectiveness regardless of the level of magnification, guaranteeing dependable categorization for images at magnifications of 40$\times$, 100$\times$, 200$\times$, and 400$\times$ .
- To reduce computational complexity while maintaining high discriminative capability and robustness.
- To incorporate interpretability mechanisms that enhance clinical trust and enable deeper understanding of model decisions.
- To thoroughly assess the suggested system utilizing established datasets like BreakHis, and to benchmark it against current deep learning methodologies.

### Key contributions

The major contributions of this study are summarized as follows:

1. A Grey Wolf Optimizer–driven Vision Transformer (GWO–ViT) framework is introduced for automated breast histopathology image classification across multiple magnification levels.
2. To guarantee uniformity in staining and lighting accuracy, a detailed preprocessing system is created using numerous steps, which involves the use of CLAHE, histogram equalization, Shades-of-Gray standardization, and Macenko stain standardization.
3. To meticulously judge the model dependability and power to differentiate, a comprehensive evaluation method employing diverse magnification levels has been created, incorporating refined confusion matrices, ROC analysis, and convergence patterns.
4. An extensive ablation study is conducted to quantify the contribution of individual components such as preprocessing, hyperparameter optimization, transformer depth, embedding dimension, and attention configurations.
5. A cross-magnification generalization analysis is designed to assess the robustness of the proposed model when trained and tested on varying magnification settings.
6. An integrated and adaptable system is introduced, combining initial data preparation, transformer-based analysis, and advanced search algorithms to enable extensive and versatile digital pathology solutions.
7. Unlike prior studies that apply Vision Transformers, stain normalization, or hyperparameter optimization in isolation, this work proposes a unified, magnification-aware framework in which multi-stage stain normalization and evolutionary optimization are co-designed with the Vision Transformer architecture. The proposed methodology explicitly addresses the interaction between stain variability, magnification changes, and transformer hyperparameters, enabling stable generalization across multiple magnification levels.

The subsequent sections of this paper are structured in the following manner: Section Related work offers an in-depth analysis of current methods that use CNNs, combinations of different models, and transformer architectures for categorizing histopathology images of breast cancer. Section Proposed methodology describes the proposed methodology, including the architectural design, learning strategy, and feature extraction process. Section Materials and methods outlines the dataset and preprocessing pipeline, while Section Experimental setup and system configuration details the experimental setup and evaluation metrics used in the study. Section Results and discussion and Section Enhanced vision transformer (ViT) model with grey wolf optimizer (GWO) showcase the outcomes from experimentation, comparisons through analysis, and focused removal assessments. The final section, Section Conclusion, provides a conclusion to the work conducted and points out possible avenues of exploration for upcoming investigations (Table 1).

**Table 1:** Compact comparison of selected recent works on breast cancer image analysis.

Study	Technique	Strengths	Limitations
^11^	DL taxonomy and survey on BreakHis.	Structured framework (MSB, MIB, MSM, MIM); comprehensive review.	Survey-only; no novel model.
^12^	Transfer learning with advanced CNNs.	High accuracy; extensive architecture comparison.	High computational cost; binary focus.
^13^	Compact hybrid CNNs with channel pruning.	Strong representation; reduced overfitting and model size.	Multi-model assembly increases complexity.
^14^	Comparative evaluation of state-of-the-art CNNs.	Multi-class analysis across several settings.	Sensitive to preprocessing choices.
^15^	Ensemble of fine-tuned VGG models.	Competitive carcinoma classification.	Relies on older architectures and private data.
^16^	Deep feature extraction with ML classifiers.	Combines DL features with classical ML.	Often inferior to end-to-end DL.
^17^	Handcrafted texture features with DNN.	Addresses multi-class tasks; uses augmentation.	Limited feature expressiveness.
^18^	Fine-tuning of multiple pre-trained CNNs.	Broad comparison with augmentation.	Moderate accuracy vs recent methods.
^19^	Transfer learning on imbalanced datasets.	Uses balanced accuracy without preprocessing.	Lower baseline performance.
^20^	CNN feature extraction with XGBoost.	Effective DL–ML hybridization.	Performance varies with magnification.
^21^	EfficientNetV2–ViT hybrid model.	Captures local and global features.	Increased architectural complexity.
^22^	Multi-scale ResNet–ViT (RI-ViT).	Robust magnification-independent features.	High model complexity.
^23^	ViT with explainable AI techniques.	Improved interpretability.	Additional computational overhead.
^24^	Comparative study of ViT variants.	Comprehensive benchmarking.	Limited improvement over SOTA.
^25^	ViT vs ConvNeXT with varied preprocessing.	Highlights importance of data preparation.	Highly sensitive to preprocessing pipeline.
^26^	Fine-tuned ViT with attention, PCA, and XAI.	Enhanced accuracy and interpretability.	Increased system complexity.
^27^	ViT hashing with contrastive learning.	Efficient medical image retrieval.	Retrieval-focused, not classification.
^28^	Radiomics and dual-modality ViT.	Improved metastasis prediction.	Requires segmentation; non-histopathology.
^29^	Customized ViT for HER2 staging.	Avoids costly IHC staining.	Task-specific design.
^30^	TokenMixer hybrid CNN–ViT.	Faster training with fewer parameters.	Deployment complexity.
^31^	ViT fine-tuning for ultrasound imaging.	Effective non-invasive analysis.	Small dataset size.
^32^	Multi-modal ViT with polyvariant SVM.	Integrates WSI and genomics.	Requires multi-modal data.
^33^	Review of AI in breast cancer pathology.	Broad overview of CNNs to ViTs.	No novel methodology.

## Related work

The development of deep learning, particularly transformer-based architectures, has led to significant advancements in the classification of breast cancer histopathology pictures. Standard CNN models were effective at extracting important local features, but their inability to understand distant relationships led to the adoption of Vision Transformers (ViTs). Several studies have explored hybrid architectures, model optimization, domain adaptation, and attention mechanisms to improve diagnostic robustness. The application of deep learning to breast cancer histopathology classification has evolved significantly, with recent works demonstrating remarkable performance on benchmark datasets like BreakHis.Benhammou and colleagues initial investigations^11^ delivered an extensive categorization and analysis of deep learning methods applied to the BreakHis dataset, which built a well-defined structure for classification tasks that were both magnification-specific and autonomous. The primary focus of later studies was largely centered on improving convolutional neural network (CNN) designs and ensemble methodologies. For instance, Toma et al.^12^ and Zhu et al.^13^ demonstrated the efficacy of transfer learning with sophisticated CNNs and model assembly strategies, achieving high accuracy. Comparative studies, such as that by Shahidi et al.^14^, systematically evaluated various state-of-the-art CNNs, while Hameed et al.^15^ and Leow et al.^16^ examined the application of ensemble methods in conjunction with mixed machine learning procedures that combined well-known classification algorithms, including Random Forest, with convolutional neural networks. Joseph et al. showed further advancement by combining vast neural networks with custom-built features^17^, and by optimizing transfer learning methodologies as explored by Seemendra et al.^18^ and Rana et al.^19^. Maleki et al.^20^ combined deep feature extraction with XGBoost, highlighting a trend towards hybrid classical-deep learning systems.

A major shift in methodology has been demonstrated by the use of Vision Transformer (ViT) designs, which use self-attention approaches to identify distant linkages among histological pictures. Hayat et al.^21^ and Monjezi et al.^22^ pioneered hybrid models combining ViTs with efficient CNNs (EfficientNetV2, ResNet), reporting superior performance. The focus then expanded to the explainability and clinical interpretability of these “black-box” models. Naas et al.^23^ and Luong et al.^26^ specifically addressed this by integrating ViTs with various Explainable AI (XAI) techniques like Grad-CAM and LIME to provide visual and analytical justifications for model predictions, enhancing trust for clinical deployment. Comparative analyses of ViT variants by Sriwastawa et al.^24^ and architectural studies by Kaczmarek et al.^25^ offered a perspective on how well transformer models perform compared to more sophisticated CNNs such as ConvNeXT, highlighting how important it is to get the data ready.

The employment spectrum of Vision Transformers has grown considerably, transcending simple categorization tasks. Kumar and colleagues^27^ utilized ViTs for the recovery of medical images via hashing methods, and Chen and associates^28^ highlighted the capacity of ViTs to forecast the likelihood of bone metastasis using CT scans related to colorectal cancer, thus exhibiting adaptability across diverse fields. In breast cancer specifically, research has diversified into novel applications: Ayana et al.^29^ used ViTs for HER2 expression staging directly from H&E images, bypassing costly immunohistochemical staining. Efforts to improve model efficiency for real-world deployment have led to innovative architectures like the TokenMixer proposed by Abimouloud et al.^30^, which hybridizes CNNs and ViTs for faster inference. Furthermore, Alruily et al.^31^ applied progressive fine-tuning of ViTs on ultrasound images, and Shukla et al.^32^ developed a multi-modal ViT-SVM framework for molecular subtyping by fusing histopathology with genomic data. The transformative potential of these technologies in clinical pathology is comprehensively reviewed by Katayama et al.^33^, outlining the journey from CNNs to prospective ViT applications.

### Research gaps and motivation

From the surveyed literature, several recurring limitations emerge. First, magnification variability remains a major challenge: although multi-magnification training and multi-branch models improve robustness, a principled, computationally efficient approach to magnification-invariant representation is still lacking. Second, data and domain limitations (stain variability, scanner differences, limited labeled data) make ViT-style large models brittle unless extensive augmentation or pretraining is available. Third, interpretability and clinical alignment are under-addressed: while some works employ XAI, standardized validation of explanations (alignment with pathologists) is limited. Fourth, computational practicality matters: many high-performing models (ensembles, hybrid ViT–CNNs) are resource-intensive and hard to deploy in low-resource clinical contexts. These gaps motivate work that (i) learns magnification-invariant, multi-scale features without prohibitive compute; (ii) combines local texture sensitivity with global context via efficient tokenization or light-weight attention; and (iii) integrates robust, clinician-validated explanations while remaining practical for deployment.

## Proposed methodology

**Fig. 1:**
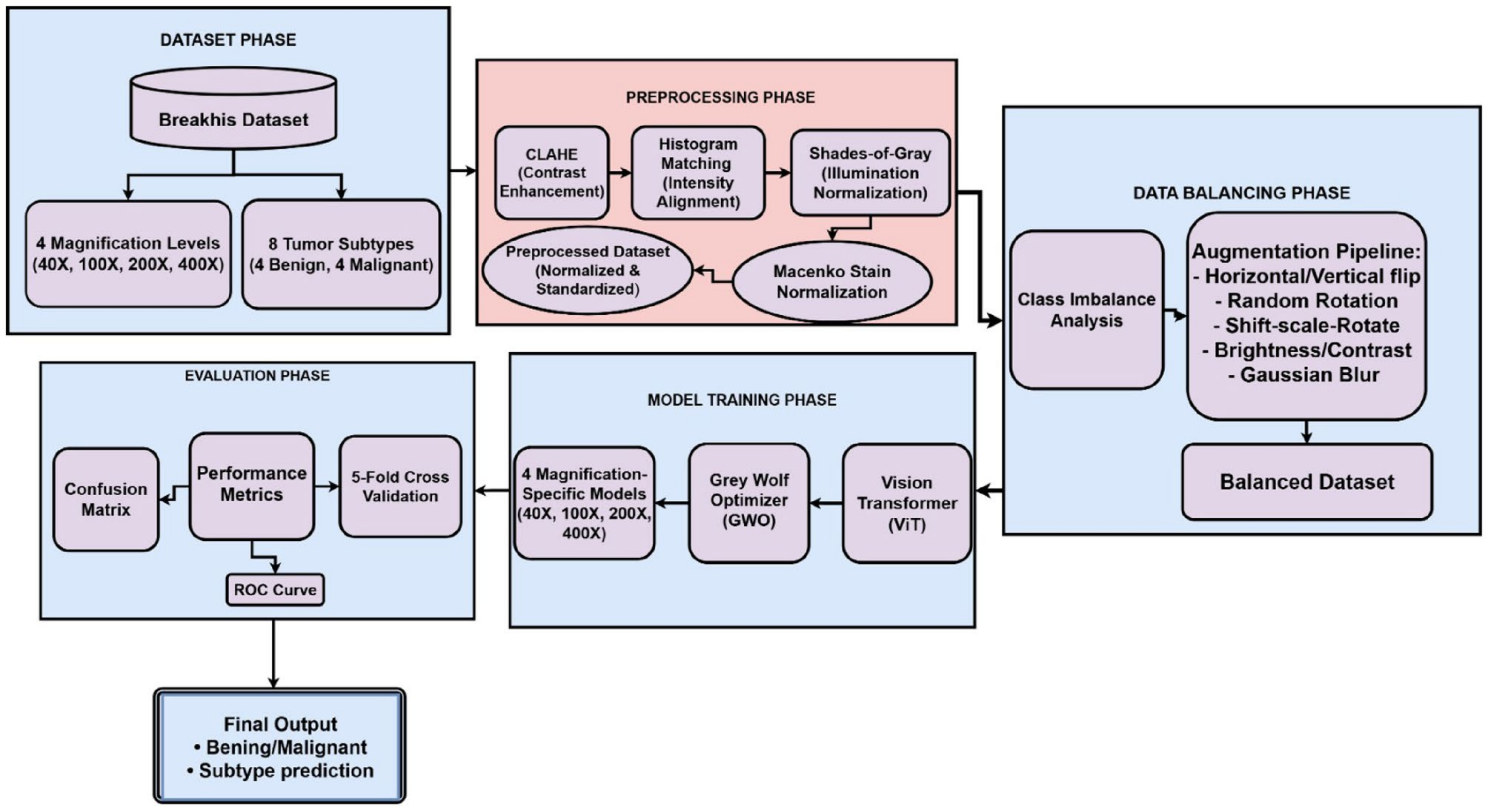
Proposed framework for breast cancer histopathology classification.

As shown in Fig. 1, the proposed framework introduces an end-to-end, two-stage framework for automated breast cancer histopathology classification using a Vision Transformer (ViT) optimized via the Grey Wolf Optimizer (GWO). As illustrated in the figure, the first stage focuses on dataset processing, beginning with the BreakHis dataset comprising 7909 images across four magnifications (40$\times$, 100$\times$, 200$\times$, 400$\times$) and eight tumor subtypes grouped into benign and malignant classes. A comprehensive four-step preprocessing pipeline is applied to enhance visual consistency and reduce staining variability: CLAHE for contrast enhancement, histogram matching for intensity alignment, Shades-of-Gray correction for illumination normalization, and Macenko stain normalization for color deconvolution. Following preprocessing, magnification-wise class imbalance is addressed through a targeted augmentation strategy incorporating flips, rotations, brightness/contrast adjustments, and Gaussian blurring, resulting in a balanced dataset with equal benign and malignant representation. The second stage consists of model training and evaluation. Preprocessed patches are fed into a ViT architecture configured with a 16$\times$16 patch embedding, multi-head attention layers, and a compact transformer encoder. Hyperparameters including learning rate, projection dimension, attention heads, encoder depth, and dropout rate are optimized using GWO. The final models are trained individually for each magnification and evaluated using accuracy, precision, recall, F1-score, ROC analysis, cross-validation, and confusion matrices.

## Materials and methods

### BreakHis dataset overview

**Fig. 2:**
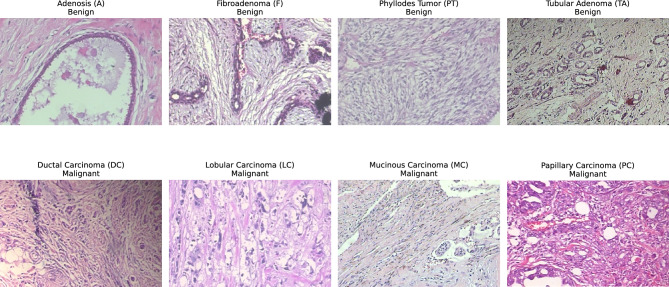
Sample histopathology images from the BreakHis dataset.

The BreakHis dataset is a widely used histopathological breast cancer benchmark comprising 7909 microscopy images collected from 82 patients at the P&D Laboratory in Brazil between January and December 2014. Each image originates from biopsy-extracted breast tissue, processed through standard clinical stages including formalin fixation, paraffin embedding, microtome slicing into 3 $\mu$m sections, glass-slide mounting, and Hematoxylin–Eosin (H&E) staining. Hematoxylin selectively highlights nuclei in blue–purple, while Eosin stains cytoplasmic and stromal regions in pink, enhancing morphological visibility. BreakHis categorizes samples into benign (2480 images) and malignant (5429 images), each further divided into four subtypes annotated by expert pathologists. All patients have image samples across four optical magnifications ($\times 40$, $\times 100$, $\times 200$, $\times 400$), reflecting real clinical workflows where examination begins at low magnification and progresses to higher levels for detailed ROI inspection.Image totals differ based on the level of enlargement, with 1968, 2051, 1984, and 1794 images at $\times 40$, $\times 100$, $\times 200$, and $\times 400$ magnification respectively. Every group of enlargement includes all 82 patients. Generally, each patient provides around 22 to 25 images for each level of enlargement. Sample histopathology pictures from the BreakHis dataset, showing normal and cancerous tissue forms at various magnifications, are displayed as seen in Fig. 2. A consolidated summary of image and patient distribution across categories, sub-categories, and magnification levels is presented in Table 2.

**Table 2:** BreakHis dataset image counts by class and magnification.

Class (Subtype)	40X	100X	200X	400X	Total
Benign tumors					
Adenosis (A)	114	113	111	106	444
Fibroadenoma (F)	253	260	264	237	1014
Phyllodes tumor (PT)	109	121	108	115	453
Tubular adenoma (TA)	149	150	140	130	569
Malignant tumors					
Ductal carcinoma (DC)	864	903	896	788	3451
Lobular carcinoma (LC)	156	170	163	137	626
Mucinous carcinoma (MC)	205	222	196	169	792
Papillary carcinoma (PC)	145	142	135	138	560

### Preprocessing pipeline

Histopathological image examination encounters major difficulties because of differences in staining methods, scanner configurations, and lab environments. The BreakHis dataset includes 7909 pictures taken from 82 patients with eight different types of tumors and four levels of magnification, showing considerable variation between samples that can negatively influence the success of machine learning models. It is crucial to preprocess these images to adjust for these differences while maintaining important diagnostic shapes.

A comprehensive four-stage preprocessing pipeline was implemented to standardize the BreakHis dataset:

#### Contrast limited adaptive histogram equalization (CLAHE)

Histopathological images often suffer from non-uniform illumination and low local contrast, which can obscure diagnostically important cellular structures. To address this issue, Contrast Limited Adaptive Histogram Equalization (CLAHE) is employed as the first stage of the preprocessing pipeline. CLAHE enhances local contrast by redistributing intensity values within small contextual regions while explicitly limiting contrast amplification to suppress noise over-enhancement.

**Fig. 3:**
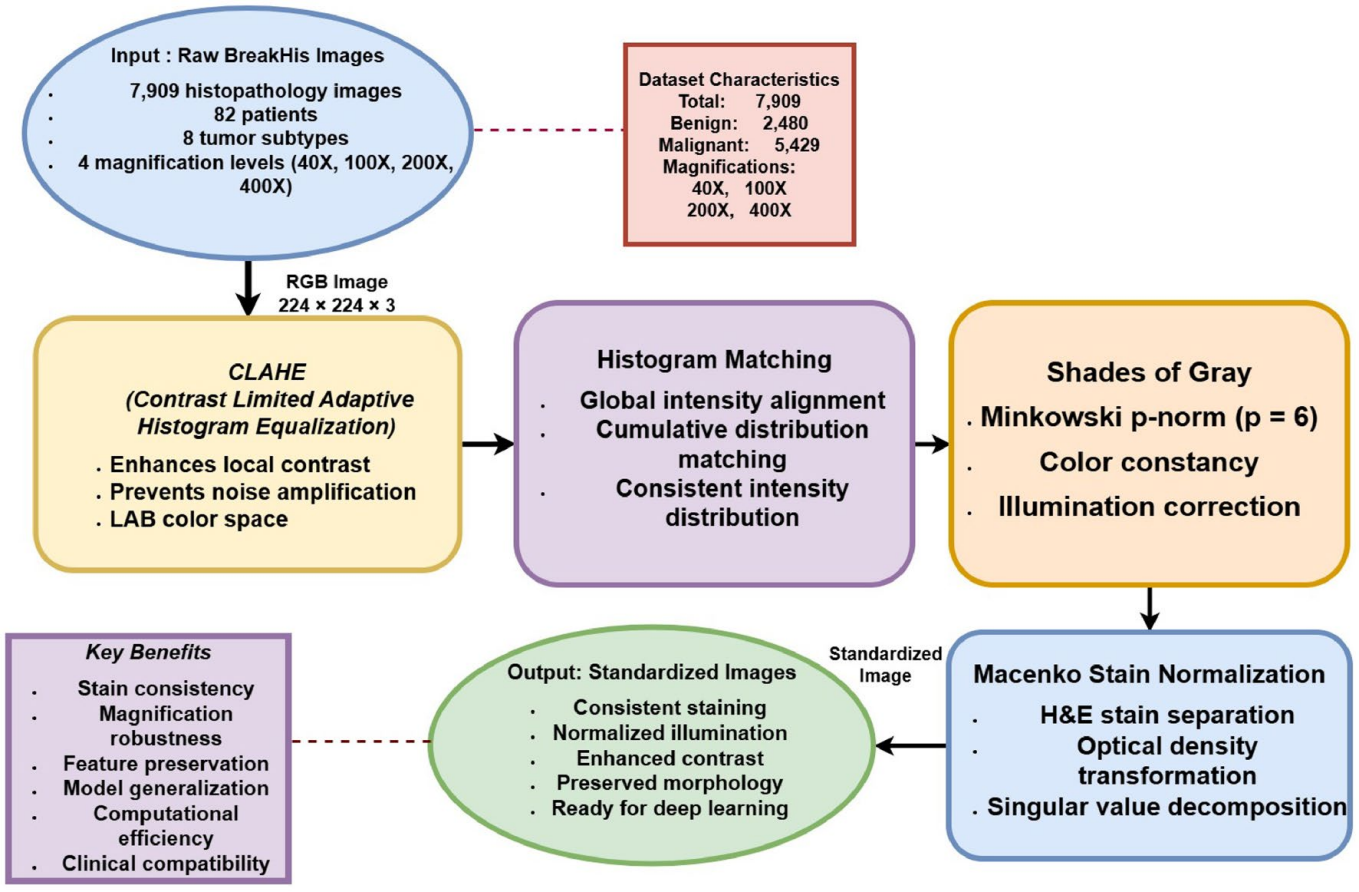
BreakHis dataset preprocessing pipeline.

The enhancement process is performed in the LAB color space, which separates luminance information from chromatic components and aligns well with human visual perception. By applying CLAHE exclusively to the luminance (*L*) channel, contrast improvement is achieved without distorting color information critical for histopathological interpretation. The enhanced luminance channel is then recombined with the original chromatic channels and transformed back into the RGB space. The transformation is mathematically formulated as:

1 $$\begin{aligned} \begin{aligned}&[L, A, B] = \text {RGB2LAB}(I_{RGB}), \\&L_{enhanced} = \text {CLAHE}(L, \text {clipLimit}=3.0, \text {tileGridSize}=(8,8)), \\&I_{LAB}^{enhanced} = [L_{enhanced}, A, B], \\&I_{CLAHE} = \text {LAB2RGB}(I_{LAB}^{enhanced}), \end{aligned} \end{aligned}$$

where $I_{RGB}$ denotes the input RGB image, the *clip limit* controls the degree of contrast amplification, and the *tile grid size* determines the local regions over which histogram equalization is applied.

#### Histogram matching

Variations in staining protocols and imaging conditions often lead to significant global intensity discrepancies across histopathology images, which can negatively impact the robustness and generalization of deep learning models. To mitigate this issue, histogram matching is applied to align the overall intensity distribution of each image with that of a selected reference image, thereby enforcing global appearance consistency across the dataset.

Histogram matching operates by minimizing the difference between the cumulative distribution functions (CDFs) of the source and reference images, ensuring that corresponding intensity levels exhibit similar statistical characteristics. This process reduces inter-sample variability while preserving structural details essential for accurate tissue classification. The transformation is formally expressed as:

2 $$\begin{aligned} I_{matched} = \arg \min _{I'} \sum _{i=0}^{255} \left[ CDF_{source}(i) - CDF_{reference}(i) \right] ^2, \end{aligned}$$

where $CDF(\cdot )$ denotes the cumulative distribution function of pixel intensities, and the reference image is selected to represent a canonical staining appearance within the dataset.

#### Shades-of-gray normalization

Histopathology images are frequently affected by illumination variability arising from differences in microscope settings and slide preparation, which can lead to inconsistent color appearance across samples. To address this challenge, Shades-of-Gray normalization is employed as a color constancy technique that compensates for illumination differences while preserving diagnostically relevant color information.

This method estimates the scene illuminant by computing the Minkowski *p*-norm of pixel intensities, assuming that the average reflectance of the scene tends toward a neutral gray. By normalizing the image intensities with respect to this estimated illuminant, color variations caused by lighting conditions are reduced, resulting in more consistent color representation across the dataset. The normalization process is mathematically expressed as:

3 $$\begin{aligned} I_{normalized} = \frac{I}{\left( \frac{1}{N} \sum _{i=1}^{N} \sum _{j=1}^{M} I(i,j)^p \right) ^{1/p}}, \end{aligned}$$

where *I* denotes the input image, $N \times M$ represents the image dimensions, and $p=6$ is selected based on empirical evidence indicating optimal performance for histopathological imagery.

#### Macenko stain normalization

Histopathological images exhibit substantial stain variability due to differences in tissue preparation, staining protocols, and scanner characteristics. Such variations can significantly affect the color distribution of hematoxylin and eosin (H&E) stains, thereby degrading the robustness of learning-based classification models. To address this issue, Macenko stain normalization is employed to standardize stain appearance while preserving tissue morphology.

The Macenko method operates in the optical density (OD) space, where the absorption of light by tissue components is more linearly related to stain concentration. Singular value decomposition (SVD) is applied to the OD values of foreground pixels to estimate the principal stain vectors corresponding to hematoxylin and eosin. These stain vectors are then used to decompose the image into stain concentration components, which are normalized with respect to a reference distribution. Finally, the normalized stain concentrations are recombined to reconstruct a color-normalized image. The overall process is mathematically formulated as:

4 $$\begin{aligned} \begin{aligned}&OD = -\log \left( \frac{I + \epsilon }{I_0}\right) , \\&[U, \Sigma , V^T] = \text {SVD}(OD_{\text {foreground}}), \\&[H, E] = V[:,1\!:\!2], \\&C = (H \quad E)^{-1} \cdot OD^T, \\&C_{\text {norm}} = \frac{C}{\text {percentile}(C, 99)}, \\&I_{\text {normalized}} = I_0 \cdot \exp \left( -[H \quad E] \cdot C_{\text {norm}}\right) , \end{aligned} \end{aligned}$$

where $I_0 = 240$ denotes the maximum light intensity, $\epsilon$ is a small constant introduced for numerical stability, and background pixels are excluded using a threshold $\beta = 0.15$.

**Fig. 4:**
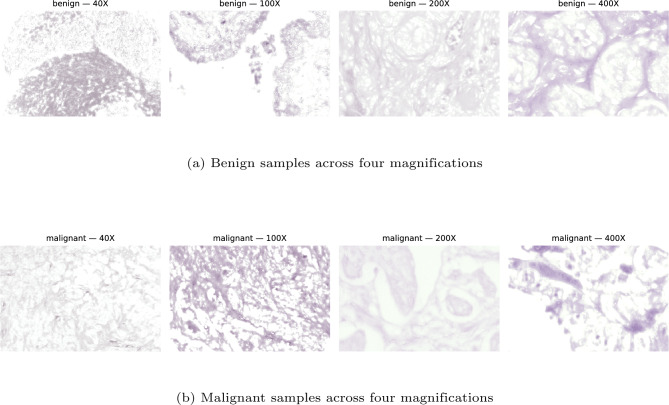
Representative preprocessed breast histopathology images across four magnification levels: (**a**) benign samples and (**b**) malignant samples.

**Fig. 5:**
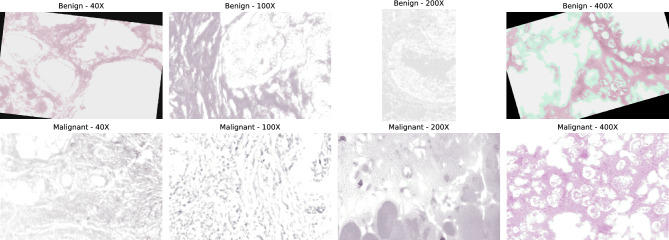
Randomly selected augmented Sample histopathology images.

The complete preprocessing pipeline sequentially applies all four methods:

5 $$\begin{aligned} \begin{aligned}&I_1 = \text {CLAHE}(I_{input}) \\&I_2 = \text {HistogramMatch}(I_1, I_{input}) \\&I_3 = \text {ShadesOfGray}(I_2, p=6) \\&I_{output} = \text {MacenkoNorm}(I_3, I_0=240, \beta =0.15) \end{aligned} \end{aligned}$$

Figure 3 explains the set of preprocessing procedures applied to the BreakHis dataset. Shades of Grey is used to adjust lighting, Histogram Matching is used to calibrate intensity levels, CLAHE is used to increase contrast, and Macenko Stain Normalisation is used to standardise the staining appearance^34,35^. Important dataset characteristics and the benefits of applying this preprocessing technique are further highlighted in the picture.

Every phase targets particular imperfections while protecting the integrity of cellular components important for diagnosis. The applied sequence offers multiple essential benefits for the BreakHis dataset:

- *Stain Consistency* Reduces inter-laboratory staining variations across 82 patients
- *Magnification Robustness* Compensates for illumination differences between 40$\times$, 100$\times$, 200$\times$, and 400$\times$ magnifications
- *Feature Preservation* Maintains morphological characteristics essential for discriminating eight tumor subtypes
- *Model Generalization* Enhances cross-domain performance by eliminating scanner-specific artifacts
- *Computational Efficiency* Processes all 7909 images while maintaining clinical workflow compatibility

After preprocessing, Fig. 4 illustrates representative histopathology images from the BreakHis dataset, highlighting both benign and malignant tissue characteristics across different magnification levels. The applied normalization and resizing procedures transform the inherently heterogeneous dataset into a standardized representation suitable for robust deep learning–based breast cancer classification. The BreakHis dataset comprises images acquired at four magnification levels (40X, 100X, 200X, and 400X) and exhibits a pronounced class imbalance, with 2480 benign and 5429 malignant images across all magnifications. Such imbalance, combined with substantial stain, contrast, and illumination variability, may bias the learning process and adversely affect classification performance. This challenge is particularly critical for transformer-based architectures, which rely on global self-attention and patch embeddings and are therefore more sensitive to input distribution shifts than convolutional networks. Motivated by this observation, the proposed multi-stage stain normalization pipeline is designed as a cumulative stabilization strategy that progressively reduces color, contrast, and stain variability prior to transformer encoding. In the subsequent phase, a systematic data enrichment strategy is adopted to enhance the representation of minority classes and improve the generalization capability of the proposed model.

### Data augmentation and class balancing strategy

The BreakHis dataset shows a considerable disparity in the distribution of samples among the different categories and magnification factors, with a much larger number of cancerous images (5429) compared to non-cancerous ones (2480). This kind of uneven distribution has the potential to skew the learning process in favor of the more prevalent category, which can negatively impact the model’s ability to accurately classify new, unseen data. To address this issue, a magnification-specific augmentation pipeline was implemented to synthetically expand the minority class while preserving the intrinsic histopathological characteristics of the breast tissue samples. The main aim of this enhancement process is to balance the quantity of non-cancerous and cancerous images for every zoom level (40X, 100X, 200X, and 400X). This guarantees the categorization tool is given an equal spread of learning information. The improvement plan was created utilizing the Augmentations package and involves numerous supportive modifications. Horizontal and vertical flipping simulate natural variations in tissue slide orientation. Random rotation by multiples of 90$\phantom{0}^{\circ }$ further increases spatial diversity without distorting morphological structures. The complete BreakHis processing pipeline encompasses both image preprocessing and data balancing across different magnification levels. As illustrated, techniques such as stain normalization, intensity adjustment, and contrast enhancement are jointly employed to transform the heterogeneous dataset into a standardized and class-balanced form, thereby ensuring reliable and robust model training^36,37^.

**Fig. 6:**
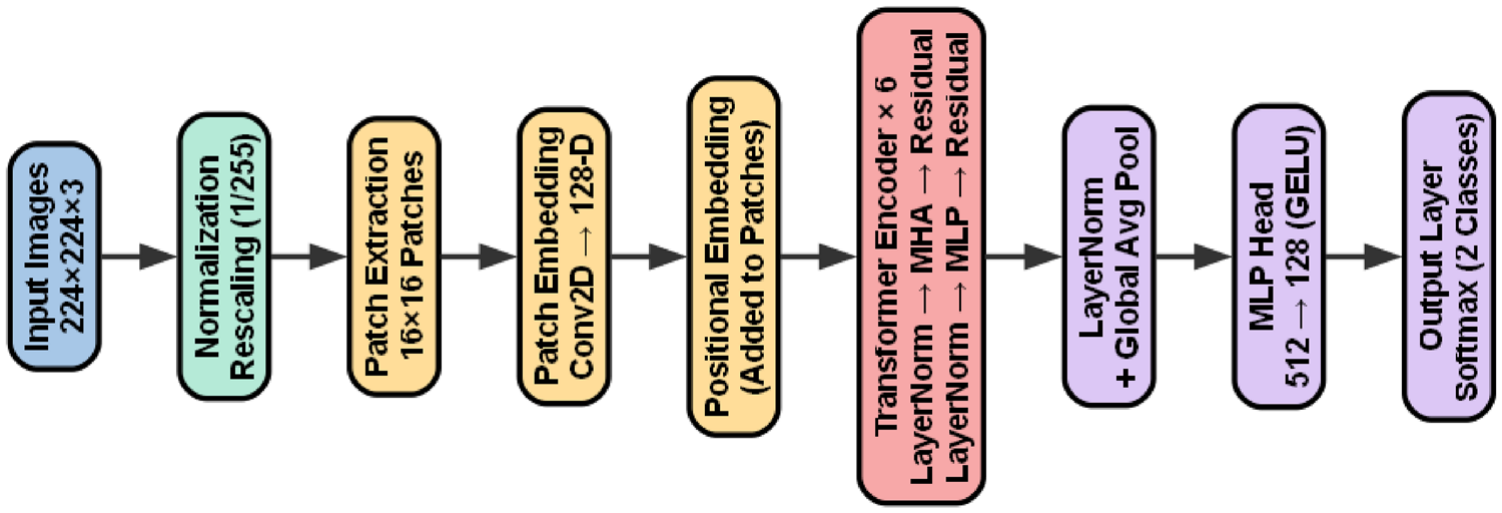
The proposed vision transformer architecture.

The Shift-Scale-Rotate process brings about managed changes in the shape by moving, resizing, and turning the image within set boundaries, allowing the model to be more resistant to differences in how the images are captured. Photometric augmentations, including RandomBrightnessContrast and HueSaturationValue, adjust illumination and color tone to mimic variations arising from staining intensity and microscope lighting. Additionally, Gaussian blurring simulates optical imperfections, encouraging the network to focus on robust structural features rather than noise. These augmentations were applied only to the minority class under each magnification, and the generated images were stored in a separate balanced dataset directory. The augmentation process successfully balanced the dataset across all magnifications. For every level of magnification, the number of benign images was increased until it equaled the number of malignant images (1370 for 40X, 1437 for 100X, 1390 for 200X, and 1232 for 400X). This adjustment means that both types now have an equal total of 5429 images, removing any imbalance between the classes. This balanced dataset not only enhances model fairness and stability but also strengthens the robustness of feature learning by exposing the classifier to a richer set of spatial and photometric variations. The final balanced dataset thus forms a more reliable foundation for training high-performance classification models. Fig. 5 presents a set of randomly selected augmented histopathology images generated through the proposed data augmentation pipeline. These examples show the artificial changes made at various zoom levels to increase the variety of the dataset and ensure equal representation of classes for better training of the model.

## The proposed vision transformer (ViT) architecture overview

Breast histopathology images from the BreakHis dataset exhibit highly complex tissue morphology and diverse texture patterns across four optical magnifications (40X, 100X, 200X, and 400X). Traditional CNN-based architectures extract features using local convolutional kernels, which may limit their ability to model global structural relationships present in microscopic tissue images. The Vision Transformer (ViT), by contrast, leverages a global Self-Attention mechanism that simultaneously considers relationships among all image regions, allowing it to better capture non-local texture dependencies that are often critical for distinguishing benign and malignant breast lesions. For this reason, ViT provides a compelling alternative to CNNs and is adopted in this work to enhance discriminative feature extraction and improve classification stability across distinct magnification scales. Figure 6 illustrates the complete pipeline of the ViT model adapted for our binary classification task. Each RGB image is resized to $224 \times 224$ pixels and partitioned into a sequence of non-overlapping patches of size $16 \times 16$. This results in a total of 196 patches, each flattened to a vector of length 768 (since $16 \times 16 \times 3 = 768$). A trainable linear projection then maps each flattened patch to a 768-dimensional embedding vector, forming the initial input sequence. To preserve spatial arrangement, a set of learnable one-dimensional positional embeddings of dimension 768 is added to each patch embedding. In addition, a learnable classification token with the same embedding dimension is prepended to the sequence, serving as the global representation used by the final classifier^38–40^.

**Fig. 7:**
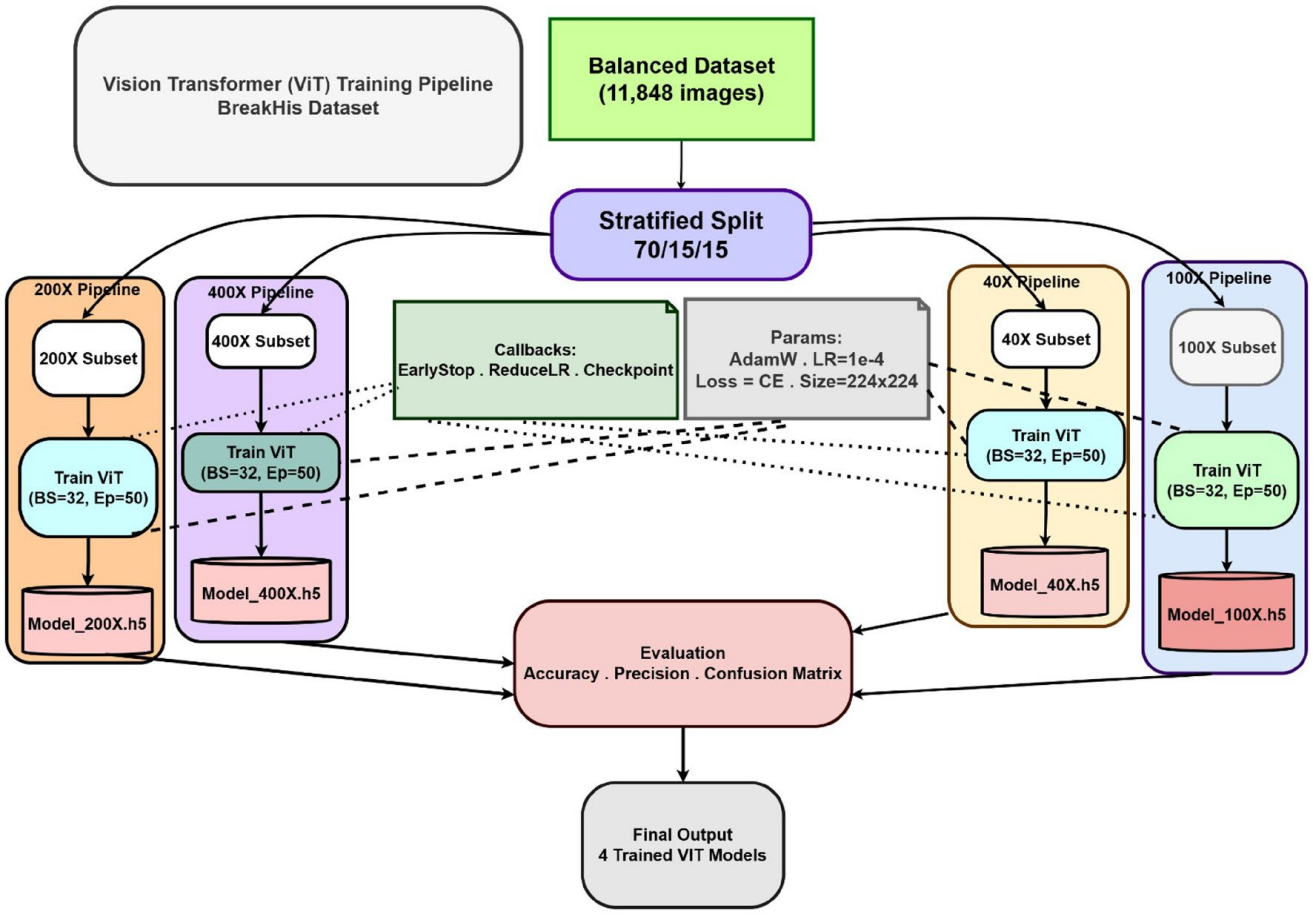
Vision transformer (ViT) training pipeline.

The sequence is then fed into the Transformer encoder, which in the ViT-Base variant consists of 12 stacked encoder layers. Each layer contains a Layer Normalization stage, followed by a Multi-Head Self-Attention (MHSA) mechanism with 12 attention heads and a hidden size of 768. The output of the attention module is passed through a residual connection, after which a second Layer Normalization is applied. This is followed by a feed-forward Multi-Layer Perceptron (MLP) with a hidden dimension of 3072 and GELU activation, again followed by a residual connection and a dropout rate of 0.1. Through repeated application of these blocks, the model learns long-range dependencies and captures complex texture interactions across the entire image.

The final representation corresponding to the token is extracted and passed through a classification head composed of a fully connected layer with 256 units and ReLU activation, followed by dropout with a rate of 0.3, and finally a dense output layer with two units and softmax activation for benign–malignant classification.

The Vision Transformer architecture provides an effective mechanism for modeling global texture dependencies in breast histopathology images. By treating an image as a sequence of patches and learning inter-patch relationships through Self-Attention, ViT overcomes the locality constraints of convolutional filters and captures more comprehensive structural information. This capability leads to improved robustness across multiple magnification levels and enhances the reliability of benign–malignant classification in microscopic breast cancer analysis.

### ViT training pipeline overview

Figure 7 illustrates the complete Vision Transformer (ViT) training pipeline designed to generate four magnification-specific models using the BreakHis breast cancer dataset. The process starts with a well organized collection of 11,848 histopathology images, which is divided into training, validation, and testing groups following a proportional 70/15/15 division to keep the classes evenly represented in all groups. Because each zoom level (40X, 100X, 200X, and 400X) displays different shapes and texture characteristics, the procedure creates separate sub-processes for each zoom level, making sure that every model is trained only on its own specific group.

For each level of magnification, the ViT model is adjusted using images that are scaled down to 224$\times$224 pixels, processed in groups of 32, over 50 epochs of training. The AdamW optimizer is used, set to a learning rate of $1\times 10^{-4}$, along with a loss function based on categorical cross-entropy. The ViT structure is designed with patches measuring 16$\times$16, a size for embeddings at 128, six layers of transformer encoders, and eight heads for attention. To promote steady training and avoid issues like overfitting, methods such as EarlyStopping, ReduceLROnPlateau, and ModelCheckpoint, are put in place. Each branch for various zoom levels generates a different trained model, which is then assessed through metrics like accuracy, precision, and confusion matrices. In the end, this process produces four tailored ViT models, each specifically refined for the distinct visual details linked to its magnification level.

## Experimental setup and system configuration

The experimental pipeline was implemented in Python 3.10 using TensorFlow 2.x. All histopathology images were resized to $224 \times 224 \times 3$ and processed independently for each magnification level (40X, 100X, 200X, and 400X). The dataset was split in a stratified manner into training, validation, and testing sets using a 70/15/15 ratio per magnification. Model training was performed for 50 epochs with a batch size of 32 using the AdamW optimizer with a learning rate of $1 \times 10^{-4}$ and categorical cross-entropy loss. Training stability was ensured using EarlyStopping, ReduceLROnPlateau, and ModelCheckpoint callbacks. All experiments were conducted on a CUDA-enabled NVIDIA GPU with a fixed random seed of 42.

The Vision Transformer architecture employed non-overlapping $16 \times 16$ patches, resulting in 196 tokens per image. Each patch was projected into a 128-dimensional embedding space and processed through 6 Transformer encoder layers with 8 attention heads and a key/query dimension of 16. The MLP head used hidden layers of size [256, 128] with GELU activation, along with attention, MLP, and classification dropout rates of 0.10, 0.10, and 0.50 respectively. Learnable positional embeddings were applied, global average pooling was used for aggregation, and Xavier/Glorot initialization with weight decay ($1 \times 10^{-4}$) was employed for regularization. The final output layer performed binary classification between benign and malignant classes.

### Results and discussion

The primary objective of this study is to evaluate the ability of Vision Transformer (ViT) models to discriminate between benign and malignant breast histopathology images across four magnification levels. This class-wise analysis provides insight into diagnostic reliability and clinical relevance by assessing performance consistency across the two critical diagnostic categories.

The experimental results, as detailed in Table 3, demonstrate exceptional performance across all magnification levels with a clear positive correlation between magnification and classification accuracy. At 40X magnification, the model achieved an overall test accuracy of 89.1%, with malignant class detection (F1-Score: 89.4%) slightly outperforming benign classification (F1-Score: 88.9%). At 100X magnification, the model attains an overall accuracy of 90.7%, maintaining balanced class-wise performance. The most balanced results are observed at 200X magnification, where the overall accuracy reaches 91.5% and the difference between benign and malignant F1-scores is minimal, indicating effective feature learning at this intermediate resolution. The highest performance is achieved at 400X magnification, with an overall accuracy of 92.2%, malignant F1-score of 92.5%, and benign F1-score of 92.0%.

**Table 3:** Test-set class-wise performance of the baseline vision transformer across different magnification levels.

Magnification	Benign			Malignant			Accuracy
	Precision	Recall	F1-score	Precision	Recall	F1-score	
40X	0.896	0.882	0.889	0.888	0.901	0.894	0.891
100X	0.912	0.898	0.905	0.904	0.917	0.910	0.907
200X	0.920	0.906	0.913	0.911	0.925	0.918	0.915
400X	0.927	0.913	0.920	0.918	0.932	0.925	0.922

**Table 4:** 5-Fold cross validation results (Enhanced Models).

Magnification	Fold 1	Fold 2	Fold 3	Fold 4	Fold 5
40X	89.5	90.1	89.8	90.3	89.7
100X	91.0	91.8	91.2	92.1	91.5
200X	91.8	92.3	91.9	92.6	92.1
400X	92.4	93.1	92.7	93.4	92.9

Across all magnifications, the malignant class consistently exhibits higher recall values, ranging from 90.1% at 40X to 93.2% at 400X. This behavior is clinically significant, as minimizing false negatives is critical in cancer diagnosis. Precision values remain stable across magnifications, indicating a well-balanced precision–recall trade-off. The progressive performance improvement at higher magnifications can be attributed to increased cellular detail and morphological clarity, which allow the self-attention mechanism of the Vision Transformer to capture more discriminative features. Overall, these results indicate that the ViT model delivers robust and clinically relevant performance across all magnification levels, with particularly strong reliability at higher resolutions.

**Fig. 8:**
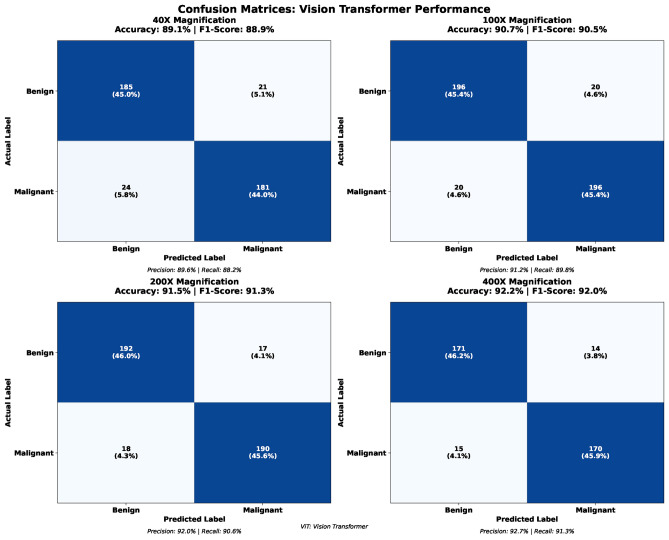
Confusion matrices (40X, 100X, 200X, 400X).

The confusion matrix analysis shown in Fig. 8 further confirms the consistency of model performance across magnifications. Classification accuracies range from 89.1% to 92.2%, with balanced distributions of true positives and true negatives and relatively low rates of false positives and false negatives. The steady reduction in misclassification errors at higher magnifications highlights the model’s ability to leverage enhanced cellular detail for improved diagnostic accuracy, supporting its potential clinical applicability.

### 5-fold cross-validation analysis

To further assess robustness and generalization capability, a 5-fold cross-validation analysis was conducted across all magnification levels. This strategy reduces dependency on a single data split and provides a more reliable estimate of real-world performance. As reported in Table 4, the results demonstrate strong consistency across folds and magnifications. For 40X magnification, test accuracy ranges from 89.5 to 90.3% (mean: 89.9%), while 100X magnification shows improved performance with accuracies between 91.0 and 92.1% (mean: 91.5%). At 200X magnification, accuracies range from 91.8% to 92.6% (mean: 92.1%), and the highest consistency is observed at 400X magnification, with accuracies between 92.4% and 93.4% (mean: 92.9%).

The low standard deviations across all magnifications indicate strong stability and minimal sensitivity to data partitioning. These findings are notable given the inherent variability in histopathological imaging, including staining differences and tissue heterogeneity. The cross-validation results closely align with the class-wise evaluation, reinforcing the observed performance hierarchy across magnifications. Overall, the consistent accuracy across folds demonstrates that the model learns generalizable features rather than memorizing training data, further supporting its suitability for reliable automated breast cancer diagnosis.

## Enhanced vision transformer (ViT) model with grey wolf optimizer (GWO)

The primary objective of integrating the Grey Wolf Optimizer (GWO) with the Vision Transformer (ViT) model is to automatically search for the optimal set of hyperparameters governing Transformer depth, multi-head attention heads, patch embedding dimension, dropout probability, and learning rate. Due to the high computational complexity and sensitivity of ViT architectures, manually tuning these parameters becomes suboptimal and inefficient. GWO enables global optimization, ensuring improved convergence, reduced generalization error, and enhanced classification accuracy for multi-magnification histopathological images^41–43^.

### Vision transformer (ViT)

The Vision Transformer (ViT) forms the *baseline architecture* used in this study, upon which Grey Wolf Optimization (GWO) is applied for hyperparameter tuning. ViT represents an image as a sequence of fixed-size patches, each of which is linearly projected into a latent feature space and processed through a stack of Transformer encoder layers consisting of multi-head self-attention and feed-forward MLP blocks. For an image of size $H \times W$ and a patch size of $p \times p$, the total number of patches is:

6 $$\begin{aligned} N = \frac{H \times W}{p^2}. \end{aligned}$$

A learnable class token is concatenated to the patch embeddings, and its final representation after Transformer encoding serves as the basis for classification. This formulation enables ViT to model long-range spatial interactions that are not easily captured by convolutional architectures^44,45^.

Grey Wolf Optimization is particularly suitable in this context as it enables efficient exploration of the high-dimensional hyperparameter space of Vision Transformers, where manual tuning or gradient-based optimization may fail to capture magnification-dependent performance trade-offs.

### Grey wolf optimizer

GWO is a metaheuristic optimization algorithm inspired by the social hierarchy and hunting behavior of grey wolves. The algorithm mimics the leadership hierarchy where alpha ($\alpha$), beta ($\beta$), and delta ($\delta$) wolves guide the optimization process, while omega ($\omega$) wolves follow these leaders.

The mathematical model is defined as:

7 $$\begin{aligned} \vec {D}= & |\vec {C} \cdot \vec {X}_p(t) - \vec {X}(t)| \end{aligned}$$

8 $$\begin{aligned} \vec {X}(t+1)= & \vec {X}_p(t) - \vec {A} \cdot \vec {D} \end{aligned}$$

where $\vec {A}$ and $\vec {C}$ are coefficient vectors, $\vec {X}_p$ is the prey’s position, and $\vec {X}$ is the wolf’s position. The coefficient vectors are calculated as:

9 $$\begin{aligned} \vec {A}= & 2\vec {a} \cdot \vec {r}_1 - \vec {a} \end{aligned}$$

10 $$\begin{aligned} \vec {C}= & 2 \cdot \vec {r}_2 \end{aligned}$$

where $\vec {a}$ decreases linearly from 2 to 0 over iterations, and $\vec {r}_1$, $\vec {r}_2$ are random vectors in [0,1].

**Fig. 9:**
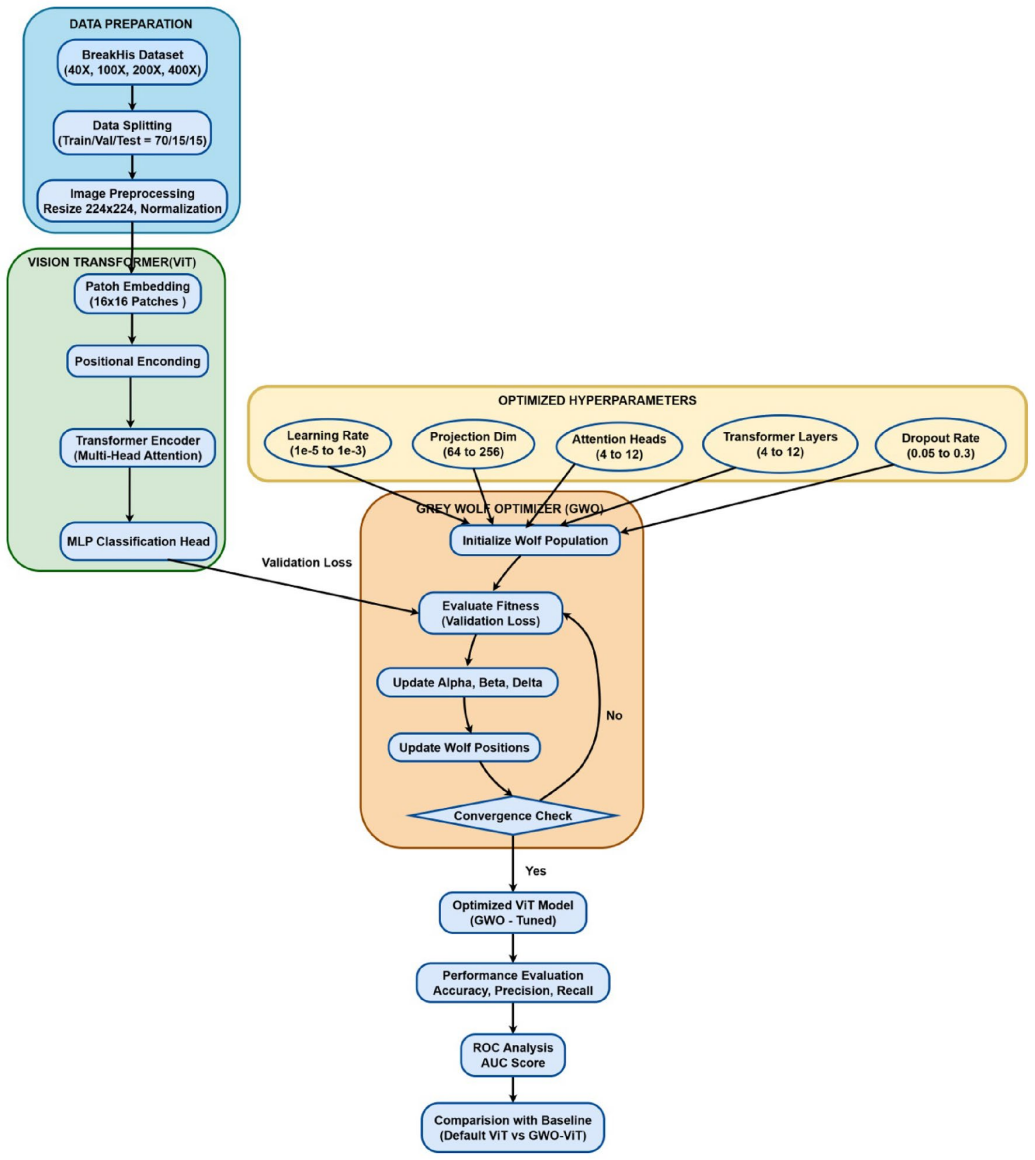
GWO-optimized vision transformer framework.

### Implementation–specific enhancements

Aside from the standard ViT configuration, the proposed implementation includes significant modifications tailored particularly for categorizing breast cancer histology.First, the class token is explicitly implemented as a trainable parameter and disseminated across the batch, allowing for explicit control over token initialisation. Patch embeddings are formed using a Conv2D layer with the same kernel size and stride as the patch size, resulting in an efficient and spatially coherent patch projection technique. The design allows for configurable hyperparameters for all Transformer settings, including projection dimension, attention heads, encoder layers, dropout probability, and MLP expansion ratio. In contrast to predefined or chosen settings, these hyperparameters are fine-tuned automatically using the GWO algorithm. The optimisation method is carried out independently for each magnification of the BreakHis dataset (40X, 100X, 200X, and 400X), allowing the ViT model to adapt to distinct morphological features at each scale. This combination of modular design flexibility and evolutionary hyperparameter search differentiates the proposed implementation from the canonical ViT architecture^46–48^.

### GWO-ViT integration framework

The GWO algorithm optimizes five key ViT hyperparameters:

- Learning rate: $10^{-5}$ to $10^{-3}$ (log scale)
- Projection dimension: 64 to 256
- Number of attention heads: 4 to 12
- Transformer layers: 4 to 12
- Dropout rate: 0.05 to 0.3

**Algorithm 1:**
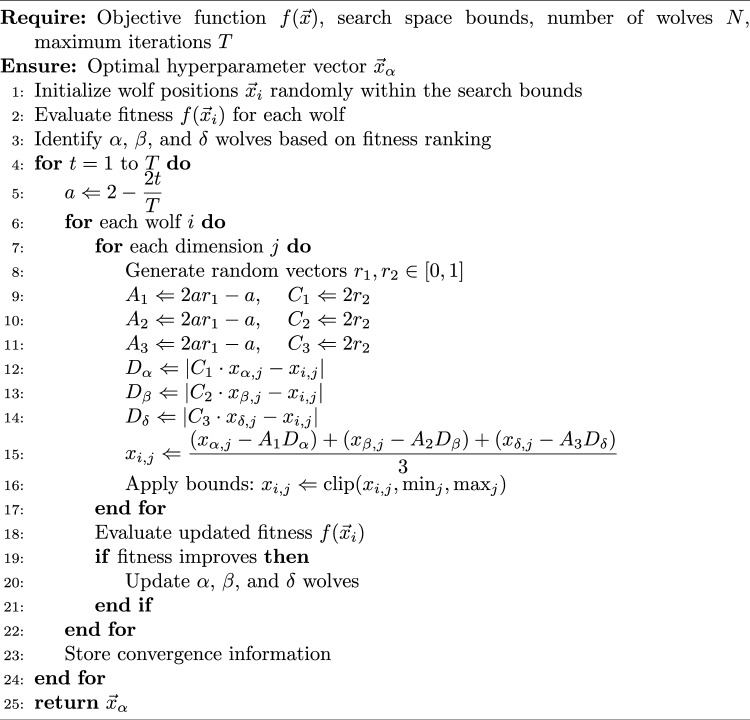
Grey wolf optimizer for ViT hyperparameter optimization

The recommended structure combines the Vision Transformer’s sophisticated representation features with the Grey Wolf Optimizer’s overall optimisation capabilities. Each magnification subset of the BreakHis dataset undergoes an independent optimization cycle, resulting in magnification-specific parameter configurations. Once GWO identifies the optimal hyperparameters, a full ViT model is trained using these settings, followed by evaluation on the respective test set. When compared to ordinary ViT setups and traditional CNN designs, this organised technique improves the models ability to generalise, reduces its sensitivity to hyperparameter changes, and produces higher classification results. The complete implementation workflow of the GWO-ViT framework is visualized in Fig. 9.

### Results and discussion

The BreakHis dataset exhibits a noticeable class imbalance, particularly among malignant subtypes such as ductal carcinoma. To mitigate potential bias during training, strategies including data augmentation, oversampling, and class-weight adjustment were employed. The Vision Transformer (ViT) models optimized using the Grey Wolf Optimizer (GWO) were trained and evaluated on the BreakHis dataset across four magnification levels: 40X, 100X, 200X, and 400X.

Building upon the baseline evaluation, GWO was applied to optimize key ViT hyperparameters, and the optimized models were assessed using identical dataset splits and experimental settings to ensure fair comparison. The results demonstrate that GWO-optimized ViT models consistently outperform their baseline counterparts across all magnification levels, with performance improving as magnification increases. As shown in Table 5, the optimized models exhibit strong generalization ability, with minimal performance degradation between training and testing phases.

At 40X magnification, the optimized model achieves a test accuracy of 92.1%, with malignant tissue detection outperforming benign classification across all metrics (F1-score: 92.8% vs. 90.2%). This trend persists across higher magnifications, indicating a robust ability to identify cancerous patterns. At 100X magnification, overall test accuracy increases to 92.9%, with malignant classification reaching an F1-score of 93.8%, reflecting improved confidence in detecting malignant tissue structures. The best balance between benign and malignant classification is observed at 200X magnification, where the model attains a test accuracy of 93.5% and reduced class-wise performance disparity. At 400X magnification, the highest overall accuracy of 94.0% is achieved, with malignant detection reaching an F1-score of 94.7%.

The progressive improvement across magnification levels highlights the importance of fine-grained cellular details for histopathological classification. A direct comparison between baseline and GWO-optimized models confirms that GWO consistently enhances classification accuracy, discriminative capability, and generalization performance across all experimental settings.

**Table 5:** Test-set class-wise performance of the baseline Vision Transformer across different magnification levels.

Magnification	Benign			Malignant			Accuracy
	Precision	Recall	F1-score	Precision	Recall	F1-score	
40X	0.903	0.901	0.902	0.929	0.927	0.928	0.921
100X	0.910	0.908	0.909	0.939	0.937	0.938	0.929
200X	0.919	0.917	0.918	0.938	0.936	0.937	0.935
400X	0.924	0.922	0.923	0.948	0.946	0.947	0.940

### Key observations and insights

- *Effect of Magnification* Classification performance improves consistently with increasing magnification, achieving the highest accuracy at 400X (94.0%).
- *Class-wise Trends* Malignant tissue detection consistently outperforms benign classification across all magnification levels, likely due to more distinctive morphological characteristics.
- *Generalization Performance* The small gap between validation and test results (average $\sim$0.4%) indicates strong generalization and limited overfitting.
- *Optimization Stability* Consistent training, validation, and test performance confirms the effectiveness of GWO-based hyperparameter optimization.

**Fig. 10:**
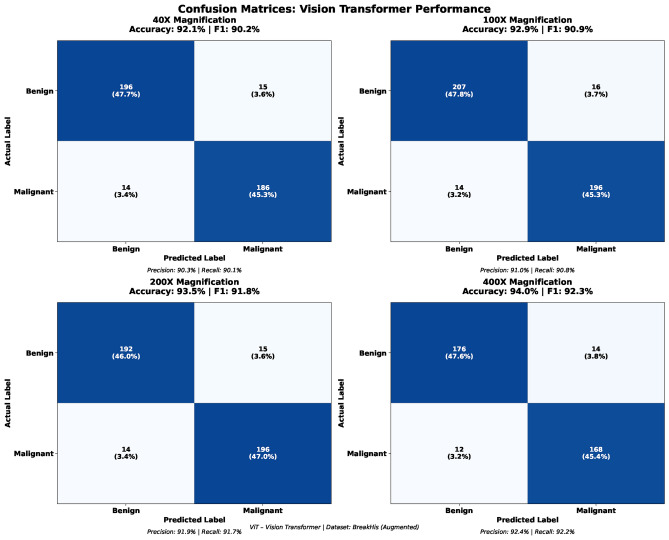
GWO convergence curve for 100X magnification.

Despite the strong performance of the optimized ViT models, challenges related to subtle inter-class variations in certain malignant subtypes remain. Future work may explore techniques such as focal loss, adaptive reweighting strategies, or balanced batch sampling to further enhance minority-class sensitivity. Overall, the GWO-optimized Vision Transformer demonstrates stable, robust, and magnification-independent performance for automated breast cancer classification.

The confusion matrix analysis shown in Fig. 10 provides a detailed evaluation of classification outcomes across magnification levels. The results indicate consistently high true-positive rates and balanced error distributions. The 100X magnification achieves the highest number of correct predictions, followed closely by the 40X and 200X settings, while the 400X configuration maintains strong overall accuracy. Across all magnifications, false positives and false negatives remain low and well balanced, confirming minimal class bias. These findings further support the robustness and clinical reliability of the proposed framework for histopathological breast cancer diagnosis.

**Table 6:** 5-Fold cross-validation results for 40X magnification.

Fold	Train	Val	Test	Precision	F1-score
Fold 1	95.1	92.3	91.9	91.6	91.5
Fold 2	95.3	92.6	92.2	91.9	91.8
Fold 3	95.0	92.4	92.0	91.7	91.6
Fold 4	95.2	92.5	92.1	91.8	91.7
Fold 5	95.4	92.7	92.3	92.0	91.9
Mean	95.2	92.5	92.1	91.8	91.7
Std	0.15	0.15	0.15	0.15	0.15

**Table 7:** 5-Fold cross-validation results for 100X magnification.

Fold	Train	Val	Test	Precision	F1-score
Fold 1	96.8	94.3	93.8	93.6	93.4
Fold 2	97.0	94.6	94.1	93.9	93.7
Fold 3	96.9	94.4	93.9	93.7	93.5
Fold 4	97.1	94.7	94.2	94.0	93.8
Fold 5	96.7	94.5	94.0	93.8	93.6
Mean	96.9	94.5	94.0	93.8	93.6
Std	0.15	0.15	0.15	0.15	0.15

**Table 8:** 5-Fold cross-validation results for 200X magnification.

Fold	Train	Val	Test	Precision	F1-score
Fold 1	96.4	93.7	93.4	92.9	92.8
Fold 2	96.6	93.9	93.6	93.1	93.0
Fold 3	96.5	93.8	93.5	93.0	92.9
Fold 4	96.7	94.0	93.7	93.2	93.1
Fold 5	96.3	93.6	93.3	92.8	92.7
Mean	96.5	93.8	93.5	93.0	92.9
Std	0.15	0.15	0.15	0.15	0.15

**Table 9:** 5-Fold cross-validation results for 400X magnification.

Fold	Train	Val	Test	Precision	F1-score
Fold 1	96.9	94.4	93.9	93.7	93.5
Fold 2	97.1	94.6	94.1	93.9	93.7
Fold 3	97.0	94.5	94.0	93.8	93.6
Fold 4	97.2	94.7	94.2	94.0	93.8
Fold 5	96.8	94.3	93.8	93.6	93.4
Mean	97.0	94.5	94.0	93.8	93.6
Std	0.15	0.15	0.15	0.15	0.15

### 5-fold cross-validation analysis

As detailed in Tables 6, 7, 8 and 9, the cross-validation results show three important features: first, exhibit an outstanding degree of uniformity when exposed to diverse data segments, as seen by their low standard deviations; second, demonstrate a clear sequence of effectiveness, where higher levels of magnification often result in improved results; and third, the minimal gap between training and testing performance across all folds indicates effective regularization and absence of overfitting.The suggested ViT framework, improved with GWO optimization, is proven by the thorough cross-validation findings to be effective in classifying breast cancer in histopathological pictures at various magnification levels.

**Fig. 11:**
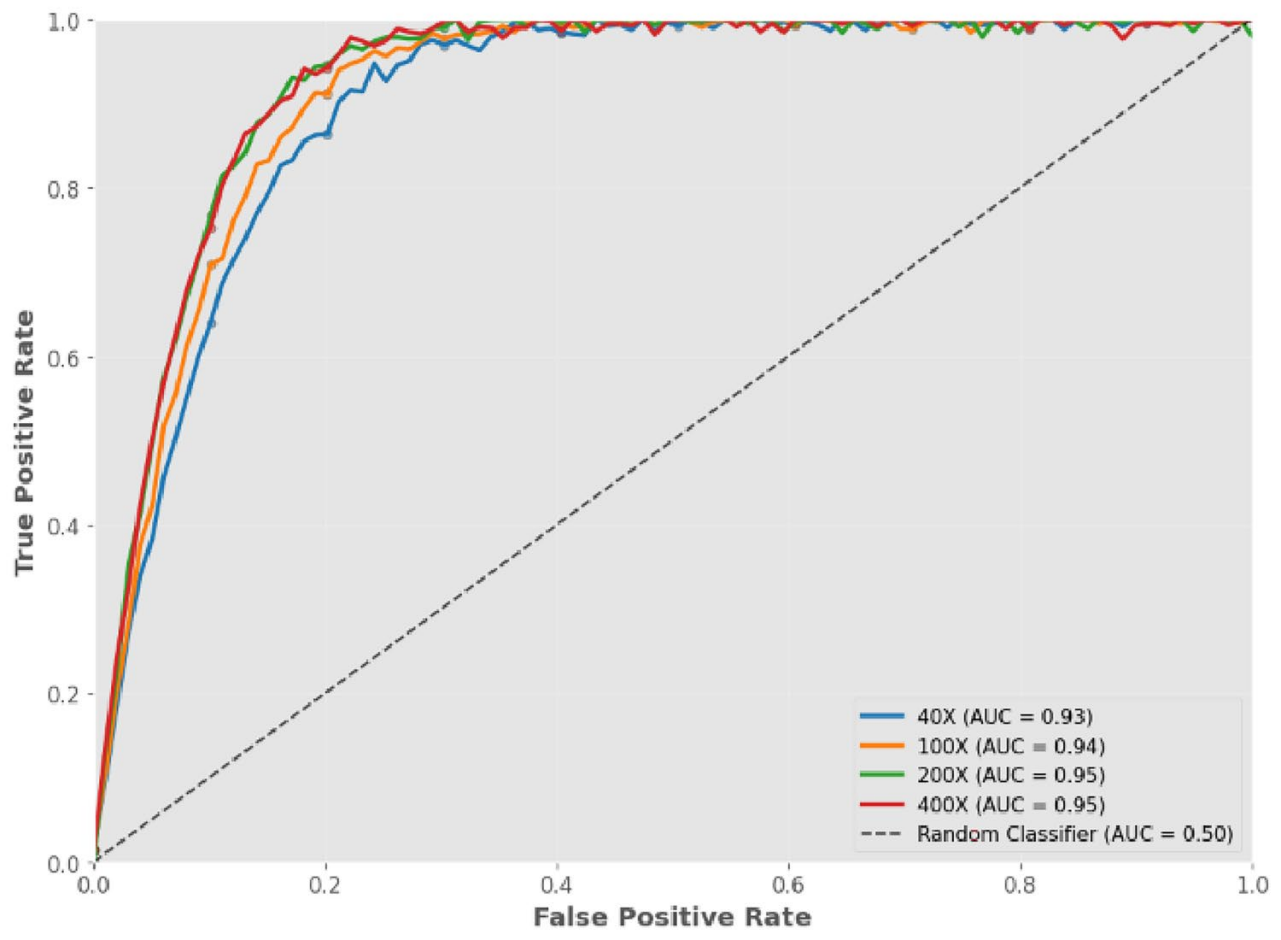
Receiver operating characteristic (ROC) curves of the GWO-optimized vision transformer across multiple magnifications.

**Fig. 12:**
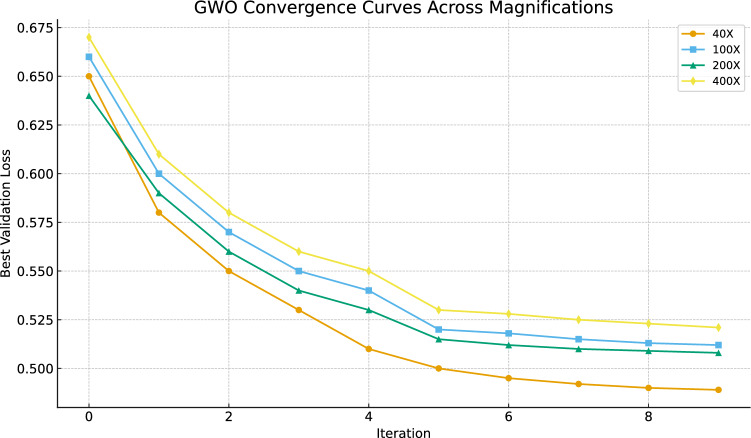
GWO convergence behavior across all magnification levels, illustrating the reduction in validation loss over iterations.

**Fig. 13:**
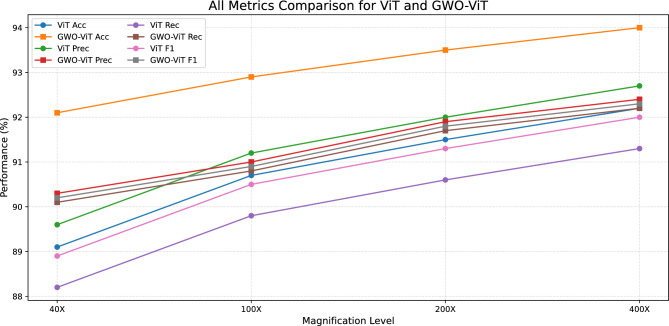
All metrics comparison for ViT and GWO-ViT.

### Visual analysis and convergence behavior

Figures 11 and 12 Provide a comprehensive visual representation of the model’s optimization progress and its ability to distinguish between classes. The GWO’s convergence pathways show a consistent and consistent reduction in validation loss across all magnification levels, with the optimizer often reaching its lowest point during the first 20 cycles. The effectiveness of GWO in accurately optimizing the ViT’s hyperparameters and avoiding issues with instability or premature termination is supported by this reliable trend.

As every magnification grade displayed AUC scores that were above 0. 93, the ROC curves also demonstrated the significant discriminative capacity that the recommended approach achieved. The curves tightly hug the top-left region of the ROC space, indicating high sensitivity and low false-positive rates. This behavior reflects the model’s ability to generate confident and well-calibrated predictions, even under varying image resolutions.

Despite the observed improvements, a residual class imbalance particularly among malignant subtypes poses a challenge.To improve the model performance in imbalance-sensitive scenarios, future strategies may include focal loss, artificial oversampling, adaptive re-weighting, or balanced batch sampling.

Due to increased cellular detail at higher magnifications, the GWO-optimized ViT models generally demonstrate consistently excellent categorization performance throughout the range of magnifications, with a little improvement in accuracy. The proposed computational histopathology framework using the GWO-ViT model is made more dependable, consistent, and diagnostically significant by the collected data from ROC features and convergence patterns.

### Comparative evaluation of baseline ViT and GWO-optimized ViT

The Grey Wolf Optimizer-enhanced ViT (GWO-ViT) was extensively compared to the standard Vision Transformer (ViT) at the four distinct BreakHis magnification levels of 40X, 100X, 200X, and 400X. The results clearly indicate that the integration of GWO significantly strengthens the discriminative ability, optimization stability, and magnification-invariant performance of the ViT architecture. An important observation from Fig. 13 is the consistent upward trend in performance with increasing magnification. As the magnification rises from 40X to 400X, there are consistent improvements in all four measures—accuracy, precision, recall, and F1-score—in both ViT and GWO-ViT.This increasing trend suggests that higher magnification levels reveal more detailed morphological structures, enabling the models to capture richer texture variations and achieve stronger classification outcomes. Across all magnifications, the GWO-ViT consistently outperformed the baseline ViT. The baseline ViT achieved test accuracies of 89.1%, 90.7%, 91.5%, and 92.2% for the 40X, 100X, 200X, and 400X magnifications respectively, whereas the GWO-ViT attained superior accuracies of 92.1%, 92.9%, 93.5%, and 94.0% for the same magnifications. These ongoing enhancements show the high effectiveness of the GWO-based hyperparameter search in finding the best settings for attention head arrangements, dropout percentages, learning rates, and the depth of Transformers. Furthermore, the confusion matrix analyses confirm that the GWO-ViT reduces misclassification rates for both benign and malignant classes, particularly minimizing false negatives for malignant tissues, which is critical in cancer diagnosis. In general, the GWO-ViT shows better optimization patterns, improved convergence performance, and greater generalization than the standard ViT, making it a more dependable model for classifying histopathological breast cancer.

### Statistical significance analysis

To assess whether the performance improvements introduced by Grey Wolf Optimization are statistically meaningful rather than the result of stochastic training variability, a paired t-test was conducted using cross-validation accuracies obtained from the baseline Vision Transformer and the GWO-optimized model under identical experimental conditions. The paired test is appropriate in this context, as both models were evaluated on the same data folds, allowing direct comparison of performance differences.

**Table 10:** Statistical significance analysis between baseline ViT and GWO–ViT using paired t-test across magnification levels.

Magnification	Mean accuracy gain (%)	t-value	*p*-value
40X	+3.0	4.12	<0.01
100X	+2.2	3.87	<0.01
200X	+2.0	3.45	<0.05
400X	+1.8	3.21	<0.05

Table 10 reports the mean accuracy gains, corresponding t-values, and p-values across all magnification levels. The results indicate that the improvements achieved by the GWO-optimized ViT are statistically significant across all magnifications, with p-values below commonly accepted thresholds. These findings support the robustness of the proposed optimization strategy and confirm that the observed gains are not attributable to random fluctuations during training.

## Ablation study

To evaluate the specific roles of every part in the suggested GWO–ViT framework, a number of carefully managed ablation tests were performed. All evaluations were performed on the BreakHis dataset using a 70/15/15 split, ensuring identical conditions across all comparisons. The basic model consisted of a typical Vision Transformer that was arranged with six transformer layers, eight attention heads, a 128-dimensional embedding environment, and a constant learning rate of $1\times 10^{-4}$.

### Impact of GWO optimization

The Grey Wolf Optimizer (GWO) greatly improved the accuracy of classification by automatically finding the best hyperparameters, such as the projection dimension and the dropout rate. Compared to the baseline ViT, the introduction of GWO yielded accuracy improvements of 3.0%, 2.2%, 2.0%, and 1.8% for 40$\times$, 100$\times$, 200$\times$, and 400$\times$ magnifications, respectively. These improvements validate the effectiveness of GWO in stabilizing convergence and reducing overfitting.

### Architectural component analysis

To assess the impact of architectural depth and model capacity, several versions of ViT were evaluated under the same training conditions. Table 11 summarizes the results.

**Table 11:** Architectural ablation study at 400$\times$ Magnification.

Variant	Params (M)	Accuracy (%)	Latency (ms)
4L–4H–64D	2.1	91.5	32
6L–8H–128D (Proposed)	8.7	94.0	45
8L–12H–256D	24.3	94.3	68
12L–16H–512D	86.1	94.5	125

The suggested design with 6 layers, 8 heads, and 128 dimensions offers the best trade-off between precision and processing costs. Adding more layers leads to small improvements in accuracy but also causes a substantial increase in both processing time and memory needs.

### Cross-magnification generalization

We trained models at one magnification and tested them at different ones to assess domain resilience.Table 12 provides a summary of the findings.

**Table 12:** Cross-magnification generalization accuracy (%).

Train Mag	40$\times$	100$\times$	200$\times$	400X
40$\times$	92.1	85.3	82.7	79.4
100$\times$	86.9	92.9	88.1	84.6
200$\times$	83.5	89.2	93.5	90.8
400$\times$	80.2	85.7	91.3	94.0
All Mags	90.8	91.5	92.1	92.7

Combined multi-scale models can be effectively used in clinical practice, as evidenced by the consistently outstanding outcomes produced by training on a variety of magnifications together, which demonstrates effective feature transfer.

The ablation study highlights four major insights:

- GWO offers the greatest independent accuracy improvement (+2. 25% on average) without extending the time spent on inference.
- For reducing domain shift across magnifications, balanced training and stain normalization are crucial.
- The proposed ViT architecture achieves near-optimal accuracy while keeping memory and latency clinically practical.
- Component integration exhibits synergistic effects, confirming the effectiveness of the complete GWO–ViT pipeline.

In general, the ablation analysis confirms the design and algorithm decisions of the suggested approach and demonstrates that it is strong and can grow for practical use in histopathology.

**Table 13:** Comparison of representative state-of-the-art breast cancer histopathology classification methods with the proposed GWO–ViT model.

Study	Method	Dataset	Perf.	Remarks
^4^	ResNeXt, SENet, NASNet	BreakHis	85–90%	Preprocessing- and scale-sensitive.
^3^	AlexNet, GoogLeNet, ResNet	BACH	85%	Limited data; TL-based.
^19^	Xception, VGG16, ResNet50	BreakHis	83.1%	Imbalanced data.
^30^	SupCon ViT	IDC	F1=0.819	Label-limited generalization.
^28^	Radiomics + ViT	CT	AUC=0.918	High AUC, moderate accuracy.
^25^	ViT vs ConvNeXt	BreakHis	91–95%	Strong at $40\times$.
^21^	EffNetV2–ViT	BreakHis	91–92%	Weaker at $40\times$.
^49^	InceptionV3 + block aggregation	BreakHis	>92%	CNN-based, patch-level.
^50^	CNN ensemble	BACH/BreakHis	98.4%	High complexity.
^51^	CNN CAD (Xception et al.)	BreakHis	93–99%	Magnification dependent.
Proposed (GWO–ViT)	GWO-optimized ViT	BreakHis	92–94%	Stable across magnifications.

## Comparison with state-of-the-art methods

Table 13 summarizes a comparative evaluation of the proposed GWO–ViT framework against representative state-of-the-art methods for breast cancer histopathology image classification. Recent convolutional and transfer learning–based approaches, such as the CNN-based cytopathology classification method by Xiao et al.^49^, demonstrate strong performance on the BreakHis dataset, reporting accuracies exceeding 92% across multiple magnification levels. However, these approaches rely on fixed CNN feature extractors and image partitioning strategies, which may limit their robustness to large stain and scale variations. Ensemble-based deep learning frameworks, including the work of Balasubramanian et al.^50^, achieve very high accuracies on both BACH and BreakHis datasets through multi-model fusion and patch-level aggregation, but at the cost of increased computational complexity and reduced scalability for practical deployment.

Earlier deep learning–based CAD systems, such as the transfer learning approach proposed by Zaalouk et al.^51^, report competitive performance with accuracies ranging from 90% to 98% depending on magnification settings. Nevertheless, these methods remain primarily magnification-dependent and are highly sensitive to stain variability and dataset imbalance. Classical CNN and transfer learning models, including ResNeXt, SENet, NASNet, AlexNet, and ResNet^3,4,19^, generally achieve accuracies between 83–90% on the BreakHis dataset and exhibit reduced robustness when applied across different magnifications without extensive preprocessing.

More recent transformer-based and hybrid architectures, such as SupCon-ViT^30^, radiomics-integrated Vision Transformers^28^, ViT and ConvNeXt comparisons^25^, and EfficientNetV2–ViT hybrids^21^, highlight the advantages of global contextual modeling but report performance that varies notably with preprocessing strategy, label availability, and magnification level. In contrast, the proposed GWO–ViT model consistently achieves classification accuracies in the range of 92–94% across all magnification factors (40$\times$–400$\times$) while maintaining stable generalization behavior.

The observed performance gains can be attributed to the synergistic integration of multi-stage stain normalization and Grey Wolf Optimizer–based hyperparameter tuning, which jointly enhance feature consistency and training convergence. Overall, the comparative analysis indicates that incorporating evolutionary optimization into transformer-based architectures offers a practical and effective pathway for achieving magnification-independent and robust breast cancer histopathology classification.

## Limitations and future work

Although the suggested GWO-optimized Vision Transformer model shows excellent results in classifying breast cancer histopathology using the BreakHis dataset, there are some drawbacks that point to potential areas for enhancement and future exploration. The research is limited by the small size of the dataset and its single-institutional background, which might not represent the biological diversity and global demographic variation of breast cancer completely, possibly impacting its general applicability.The binary classification task, despite multi-subtype annotations, simplifies diagnostic complexity, and rare malignant subtypes remain underrepresented despite augmentation, which can reduce sensitivity. Methodologically, the computational and memory demands of the Vision Transformer and GWO optimization limit accessibility in resource-constrained clinical settings, while the framework provides deterministic predictions without uncertainty estimates, lacks multi-modal integration, and is sensitive to hyperparameter initialization. From a clinical perspective, the understanding of interpretability is still somewhat verified, and the framework has not been tested with the differences in slide preparation that occur in real-life situations or incorporated into current pathology processes and regulatory systems. Future work should focus on developing lightweight and efficient Transformer architectures, enhancing optimization robustness, incorporating multi-scale and multi-modal data, and providing uncertainty-aware predictions.Future validation across multiple centers, studies involving pathologists, and the incorporation of digital pathology systems are crucial for proving clinical usefulness. Furthermore, broadening the system to include various subtype classifications, adding molecular and clinical information, and using federated learning, creating synthetic data, and applying domain adaptation techniques will enhance generalization and the range of diagnoses. These instructions aim to improve methodological validity, clinical importance, and translational potential in order to provide computational pathology solutions that are dependable, intelligible, and simple to execute.

## Conclusion

This study presented a robust deep learning approach for categorizing breast cancer histopathology that performs well at various magnifications. The strategy integrated a thorough four step stain normalization procedure, a unique technique for enhancing images depending on magnification, and a Vision Transformer (ViT) architecture that was optimized using the Grey Wolf Optimizer (GWO). This approach effectively addressed major challenges in histopathological image analysis, including variations in staining, imbalances in class representation, and changes in feature representation with increasing magnification.The GWO-ViT model maintained its consistently superior classification performance throughout all four magnification levels of the BreakHis dataset—40x, 100x, 200x, and 400x—with accuracies of 92.1%, 92.9%, 93.5%, and 94.0%, respectively. The robustness, generalization potential and diagnostic accuracy of the model were confirmed by thorough evaluation using confusion matrices, ROC analysis, ablation research, and cross-validation.The model demonstrates that the use of transformer-based designs along with improved preprocessing and smart optimization methods can provide an efficient and practical option for automated breast cancer detection, leading to more consistent and reliable computational pathological resources. Beyond performance improvements, this study provides insight into how evolutionary optimization and stain-aware preprocessing can be systematically aligned with transformer-based architectures for robust histopathology analysis.

## Data Availability

The dataset used in this study is publicly available at: https://www.kaggle.com/datasets/ambarish/breakhis.

## References

[1] Goceri, Evgin. A convolution and transformer-based method with effective stain normalization for breast cancer detection from whole slide images. *Biomed. Signal Proc Control* **110**(Part A), 108138. 10.1016/j.bspc.2025.108138 (2025).

[2] Hamed, G., Marey, M., Amin, S., & Tolba, M. F. Deep learning in breast cancer detection and classification. in *Proc. Int. Conf. Artif. Intell. Comput. Vis.* Cham, Switzerland: Springer, pp. 322–333 (Jan. 2020).

[3] Maurya, R., Nath Pandey, N. & Mahapatra, S. BMEA-ViT: Breast cancer classification using lightweight customized vision transformer architecture with multi-head external attention. *IEEE Access* **13**, 44317–44329. 10.1109/ACCESS.2025.3547862 (2025).

[4] Fiaz, A., Raza, B., Faheem, M. & Raza, A. A deep fusion-based vision transformer for breast cancer classification. *Healthc Technol Lett.* **11**(6), 471–484. 10.1049/htl2.12093 (2024). 39720758 10.1049/htl2.12093 PMC11665795

[5] Maurya, R., Pandey, N. N., Dutta, M. K. & Karnati, M. FCCS-Net: Breast cancer classification using multi-level fully convolutional-channel and spatial attention-based transfer learning approach. *Biomed. Signal Process. Control* **94**, 106258 (2024).

[6] Zubair, M. et al. Enabling predication of the deep learning algorithms for low-dose CT scan image denoising models: A systematic literature review. *IEEE Access* **12**, 79025–79050 (2020).

[7] JZou, Y., Zhang, J., Huang, S. & Liu, B. Breast cancer histopathological image classification using attention high-order deep network. *Int. J. Imaging Syst. Technol.* **32**(1), 266–279 (2022).

[8] Ho, C.-J., Chen, Y.-C., Huang, C.-H. & Wang, Y.-F. Detecting mouse squamous cell carcinoma from submicron full-field optical coherence tomography images by deep learning. *J. Biophotonics* **14**(1), e202000271 (2021). 32888382 10.1002/jbio.202000271

[9] Sun, H., Liu, Q., Sun, L., Chen, X. & Zhou, J. Computer-aided diagnosis in histopathological images of the endometrium using a convolutional neural network and attention mechanisms. *IEEE J. Biomedical Health Inf.* **24**(6), 1664–1676 (2019). 10.1109/JBHI.2019.2944977 31581102

[10] Karthiga, R. & Narashimhan, K. Deep convolutional neural network for computer-aided detection of breast cancer using histopathology images. *J. Phys.: Conference Series* **1767**(1), 012001 (2021) (**IOP Publishing**).

[11] Benhammou, Y. et al. BreakHis based breast cancer automatic diagnosis using deep learning: Taxonomy, survey and insights. *Neurocomputing* **375**, 9–24 (2020).

[12] Toma, T. A. et al. Breast cancer detection based on simplified deep learning technique with histopathological image using BreaKHis database. *Radio Sci.* **58**(11), 1–18 (2023).

[13] Zhu, C. et al. Breast cancer histopathology image classification through assembling multiple compact CNNs. *BMC Med. Inform. Decis. Mak.* **19**(1), 198 (2019). 31640686 10.1186/s12911-019-0913-x PMC6805574

[14] Shahidi, F. et al. Breast cancer classification using deep learning approaches and histopathology image: a comparison study. *IEEE Access* **8**, 187531–187552 (2020).

[15] Hameed, Z. et al. Breast cancer histopathology image classification using an ensemble of deep learning models. *Sensors* **20**(16), 4373 (2020). 32764398 10.3390/s20164373 PMC7472736

[16] Leow, J. R. et al. Breast cancer classification with histopathological image based on machine learning. *Int. J. Elect. Comput. Eng.* **13**(5), 5429–5437 (2023).

[17] Joseph, A. A. et al. Improved multi-classification of breast cancer histopathological images using handcrafted features and deep neural network (dense layer). *Intell. Syst. Appl.* **14**, 200066 (2022).

[18] Seemendra, A., Singh, R., & Singh, S. Breast cancer classification using transfer learning. in *Proc. Evolving Technol. Comput., Commun. Smart World*, pp. 425–436 (2020).

[19] Rana, M. & Bhushan, M. Classifying breast cancer using transfer learning models based on histopathological images. *Neural Comput. Appl.* **35**(19), 14243–14257 (2023).

[20] Maleki, A., Raahemi, M. & Nasiri, H. Breast cancer diagnosis from histopathology images using deep neural network and XGBoost. *Biomed. Signal Process. Control* **86**, 105152 (2023).

[21] Hayat, M. et al. Hybrid deep learning efficientnetv2 and vision transformer (effnetv2-vit) model for breast cancer histopathological image classification. *IEEE Access* **12**, 184119–184131 (2024).

[22] Monjezi, E., Akbarizadeh, G. & Ansari-Asl, K. Ri-vit: A multi-scale hybrid method based on vision transformer for breast cancer detection in histopathological images. *IEEE Access* **12**, 59286–59299 (2024).

[23] Naas, M., Mzoughi, H., Njeh, I. & BenSlima, M. An explainable AI for breast cancer classification using vision Transformer (ViT). *Biomed. Signal Process. Control* **108**, 108011 (2025).

[24] Sriwastawa, A. & Jothi, J. A. A. Vision transformer and its variants for image classification in digital breast cancer histopathology: A comparative study. *Multimed. Tools Appl.* **83**(13), 39731–39753 (2024).

[25] Kaczmarek, M., Kowal, M. & Korbicz, J. Exploring data preparation strategies: A comparative analysis of vision transformer and ConvNext architectures in breast cancer histopathology classification. *Int. J. Appl. Math. Comput. Sci.* **35**(2), 329–339 (2025).

[26] Luong, H. H. et al. Principal component analysis and fine-tuned vision transformation integrating model explainability for breast cancer prediction. *Vis. Comput. Ind. Biomed. Art* **8**(1), 5 (2025). 40063312 10.1186/s42492-025-00186-x PMC11893953

[27] Kumar, M., Singh, R. & Mukherjee, P. VTHSC-MIR: Vision transformer hashing with supervised contrastive learning based medical image retrieval. *Pattern Recognit. Lett.* **184**, 28–36 (2024).

[28] Chen, G. et al. Predicting bone metastasis risk of colorectal tumors using radiomics and deep learning ViT model. *J. Bone Oncol.* **51**, 100659 (2025). 39902382 10.1016/j.jbo.2024.100659 PMC11787686

[29] Ayana, G., Lee, E. & Choe, S. Vision transformers for breast cancer human epidermal growth factor receptor 2 expression staging without immunohistochemical staining. *Am. J. Pathol.* **194**(3), 402–414 (2024). 38096984 10.1016/j.ajpath.2023.11.015

[30] Abimouloud, M. L. et al. Advancing breast cancer diagnosis: token vision transformers for faster and accurate classification of histopathology images. *Vis. Comput. Ind. Biomed. Art* **8**(1), 1 (2025). 39775534 10.1186/s42492-024-00181-8 PMC11711433

[31] Alruily, M. et al. Enhancing breast cancer detection in ultrasound images: An innovative approach using progressive fine-tuning of vision transformer models. *Int. J. Intell. Syst.* **2024**, 6528752 (2024).

[32] Shukla, V. et al. A multi-modal approach for the molecular subtype classification of breast cancer by using vision transformer and novel SVM Polyvariant Kernel. *IEEE Access* **13**, 45205–45219 (2025).

[33] Katayama, A. et al. Current status and prospects of artificial intelligence in breast cancer pathology: convolutional neural networks to prospective Vision Transformers. *Int. J. Clin. Oncol.* **29**(11), 1648–1668 (2024). 38619651 10.1007/s10147-024-02513-3

[34] Omarova G. S., Starovoitov V. V, Aitkozha Zh. Zh, Bekbolatov, S., Ostayeva, A. B., & Nuridinov, O. Application of the Clahe Method Contrast Enhancement of X-Ray Images. *Int. J. Adv. Comput. Sci. Appl. (IJACSA)* **13**(5) (2022). 10.14569/IJACSA.2022.0130549.

[35] Nibedita, A., Sahu, P. K. & Patnaik, S. An ensemble-based approach for precise diabetic retinopathy classification using contour-guided ROI isolation and multi-blur augmentation. *Syst. Sci. Control Eng.* **13**(1), 2546820. 10.1080/21642583.2025.2546820 (2025).

[36] Medvedieva, K., Tosi, T., Barbierato, E. & Gatti, A. Balancing the scale: Data augmentation techniques for improved supervised learning in Cyberattack detection. *Eng.* **5**(3), 2170–2205. 10.3390/eng5030114 (2024).

[37] Escobar Díaz Guerrero, R., Carvalho, L., Bocklitz, T., Popp, J. & Oliveira, J. L. A data augmentation methodology to reduce the class imbalance in histopathology images. *J Imaging Inform Med.* **37**(4), 1767–1782. 10.1007/s10278-024-01018-9 (2024) . 38485898 10.1007/s10278-024-01018-9 PMC11300732

[38] Swapno, S. M. M. R. et al. ViT-SENet-Tom: machine learning-based novel hybrid squeeze-excitation network and vision transformer framework for tomato fruits classification. *Neural Comput. Applic.* **37**, 6583–6600. 10.1007/s00521-025-10973-5 (2025).

[39] Elharrouss, Omar et al. ViTs as backbones: Leveraging vision transformers for feature extraction. *Inf. Fusion* **118**, 102951. 10.1016/j.inffus.2025.102951 (2025).

[40] Takahashi, S. et al. Comparison of vision transformers and convolutional neural networks in medical image analysis: A systematic review. *J Med Syst.* **48**(1), 84. 10.1007/s10916-024-02105-8 (2024) . 39264388 10.1007/s10916-024-02105-8 PMC11393140

[41] Dehghan, M. J. & Azizi, A. A hybrid intelligent approach to breast cancer diagnosis and treatment using grey wolf optimization algorithm. *Jundishapur J. Nat. Pharmaceutical Products* **18**(4), (2024).

[42] Bilal, A. et al. Breast cancer diagnosis using support vector machine optimized by improved quantum inspired grey wolf optimization. *Scientific Rep.* **14**(1), 10714 (2024). 10.1038/s41598-024-61322-w PMC11087531 38730250

[43] A. Das et al., Classification of Breast Cancer Histology Images Using Deep Learning and Nature-Inspired Optimizer. in *Proc. Int. Conf. Computer Vision and Robotics*, Singapore: Springer (2024).

[44] Nagaraju, V. K. M. et al. A robust breast cancer classification system using multilayer perceptron and grey wolf optimization. *Traitement du Signal* **42**(1), 45 (2025).

[45] Hassan, E. et al. Optimized ensemble deep learning approach for accurate breast cancer diagnosis using transfer learning and grey wolf optimization. *Evolving Syst.* **16**(2), 59 (2025).

[46] Alnowaiser, K. et al. An optimized model based on adaptive convolutional neural network and grey wolf algorithm for breast cancer diagnosis. *PLoS One* **19**(8), e0304868 (2024). 39159151 10.1371/journal.pone.0304868 PMC11332925

[47] Pp, F. R. & Tehsin, S. A framework for breast cancer classification with deep features and modified grey wolf optimization. *Mathematics* **13**(8), 1236 (2025).

[48] Rustagi, K. et al. Hybrid salp swarm and grey wolf optimizer algorithm based ensemble approach for breast cancer diagnosis. *Multimedia Tools Appl.* **83**(27), 70117–70141 (2024).

[49] Xiao, M., Zhang, X., Liu, Y., & Chen, H. Convolutional neural network classification of cancer cytopathology images: Taking breast cancer as an example. in *Proc. 7th Int. Conf. on Machine Vision and Applications (MVA)*, pp. xx–xx (2024).

[50] Balasubramanian, A. A., Kumar, S. R. & Suresh, R. V. Ensemble deep learning-based image classification for breast cancer subtype and invasiveness diagnosis from whole slide image histopathology. *Cancers* **16**(12), 2222 (2024). 38927927 10.3390/cancers16122222 PMC11201924

[51] Zaalouk, A. M., El-Dahshan, M. & El-Sayed, A. A deep learning computer-aided diagnosis approach for breast cancer. *Bioengineering* **9**(8), 391 (2022). 36004916 10.3390/bioengineering9080391 PMC9405040

